# Linking Gas-Phase and Solution-Phase Protein Unfolding via Mobile Proton Simulations

**DOI:** 10.1021/acs.analchem.2c03352

**Published:** 2022-11-09

**Authors:** Charles Eldrid, Tristan Cragnolini, Aisha Ben-Younis, Junjie Zou, Daniel P. Raleigh, Konstantinos Thalassinos

**Affiliations:** † School of Biological Sciences, University of Southampton, SouthamptonSO16 1BJ, U.K.; ‡ Institute of Structural and Molecular Biology, Division of Bioscience, University College London, LondonWC1E 6BT, U.K.; § Institute of Structural and Molecular Biology, Birkbeck College, University of London, LondonWC1E 7HX, U.K.; ∥ Department of Chemistry, Stony Brook University, 100 Nicolls Rd., Stony Brook, New York11794, United States

## Abstract

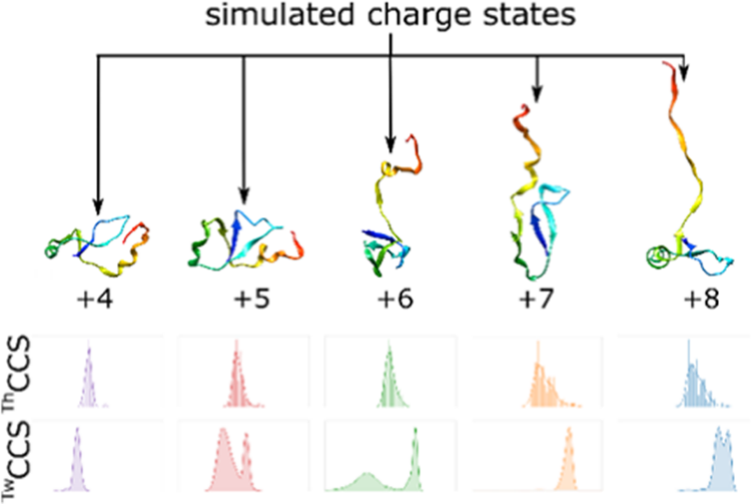

Native mass spectrometry coupled to ion mobility (IM-MS) combined with collisional activation (CA) of ions in the gas phase (*in vacuo*) is an important method for the study of protein unfolding. It has advantages over classical biophysical and structural techniques as it can be used to analyze small volumes of low-concentration heterogeneous mixtures while maintaining solution-like behavior and does not require labeling with fluorescent or other probes. It is unclear, however, whether the unfolding observed during collision activation experiments mirrors solution-phase unfolding. To bridge the gap between *in vacuo* and *in-solution* behavior, we use unbiased molecular dynamics (MD) to create *in silico* models of *in vacuo* unfolding of a well-studied protein, the N-terminal domain of ribosomal L9 (NTL9) protein. We utilize a mobile proton algorithm (MPA) to create 100 thermally unfolded and coulombically unfolded *in silico* models for observed charge states of NTL9. The unfolding behavior *in silico* replicates the behavior in-solution and is in line with the *in vacuo* observations; however, the theoretical collision cross section (CCS) of the *in silico* models was lower compared to that of the *in vacuo* data, which may reflect reduced sampling.

The study of protein unfolding is essential for defining protein stability and provides important insight into protein aggregation in protein misfolding diseases^1^ such as α1-antitrypsin deficiency,^2^ transthyretin amyloidosis,^3^ and β2 microglobulin amyloidosis.^4^ Many techniques have been developed to study protein unfolding, including circular dichroism (CD),^5^ nuclear magnetic resonance (NMR) spectroscopy,^6^ electron paramagnetic resonance (EPR) spectroscopy,^7^ fluorescence-based methods, and native mass spectrometry (MS) coupled to ion mobility (IM).^8−12^ They all offer advantages and disadvantages, but with the exception of single-molecule methods, solution-phase methods generally have difficulties characterizing heterogeneous mixtures. While powerful, single-molecule methods require labeling with often large and bulky fluorophores, MS-based methods are particularly well suited to probing heterogeneous mixtures and have the advantage that small amounts of material are required, and modifications or labeling with probes is not required.

Native MS is widely used to probe the native-like state of proteins *via* soft-ionization techniques, such as nanoelectrospray ionization (nESI)^13−15^ and enables measurement of the global protein fold,^16^ ligand binding,^17^ subunit composition of protein complexes^18^ and proteoforms.^19^ Ion mobility (IM) coupled to MS (IM-MS) adds an extra layer of information by separating isobaric protein ions *via* their 3-dimensional shape or collision cross section (CCS). IM functions by passing analyte ions through an inert buffer gas in a drift region. Ions of the same *m*/*z* but different conformation will be separated as more extended ions will experience a greater number of collisions with the buffer gas and so traverse the drift region more slowly than a more compact ion.^20,21^

IM-MS has been used to study protein dynamics and^18,22,23^ domain organization^24^ and to investigate the structural dynamics of disordered proteins.^25^ Protein ions can be collisionally activated (CA) by increasing the energy by which they are introduced into the mobility region. They are often, but not always,^26^ unfolded in a process called collision-induced unfolding (CIU). CA can give information on distinguishing features of monoclonal antibodies,^12,27,28^ the number of domains within a protein,^11^ and the thermal stability imparted by ligand binding.^17^ Adding an extra stage of IM separation (tandem-IM), to select out particular conformers, allows even greater disambiguation of the unfolding pathways of proteins by selecting precursor ions.^21,26,29,30^

While collision activation is able to give important information about unfolding and native-like states that are retained in the gas phase^31−33^ (*in vacuo*), it is not known whether gas-phase unfolding is comparable to unfolding in-solution (*in-solution*). As CCS is an inherently low-resolution structural parameter, complementary techniques are required to create a structural model of the unfolded protein *in vacuo*. Molecular dynamics (MD) simulations (*in silico*) are uniquely positioned to do so and can be coupled with IM-MS data; they provide atomistic detail, which is complementary to IM and can be benchmarked *via* comparison of experimental and theoretical CCS values.^34^

Simulations which replicate the gas-phase environment inside an IM drift cell are not as straightforward as simply simulating proteins without bulk solvent. Without the intramolecular coulombic repulsion brought about by charging, the extended states of gas-phase proteins are liable to collapse.^35,36^ In positive nESI, charged sites occur on exposed ionizable sites, such as the N-terminus, lysines, arginines, and histidines and can migrate between these sites, maintaining dynamic equilibrium.^37,38^ To account for these effects, frameworks which allow simulation of the dynamic protonation states of proteins in the gas-phase behavior have been developed.^39−42^

It is still unclear if unfolding *in vacuo* mimics the unfolding process *in-solution*, and it is also difficult to fully validate the assignment of *in vacuo* structures from *in silico* methods. To critically validate the methodology, a protein system for which there is detailed *in-solution* unfolding data must be used. To this end, we chose the N-terminal region of the ribosomal L9 protein from *Geobacillus stearothermophilus* as a model system.^43,44^ The L9 protein comprises two distinct globular domains joined by an α-helical linker in a “dumbbell” shape. Both the N-terminal (NTL9)^45−51^ and C-terminal (CTL9)^52−55^ domains are stable in isolation and fold cooperatively. Each has had its *in-solution* unfolding explored in detail through fluorescence studies, CD, and NMR line-shape analysis. The structure of the N-terminal domain has been determined *via* X-ray crystallography and adopts the same fold in isolation as in the intact protein.^55^ NTL9 is one of the simplest examples of the split β–α–β motif. The fold consists of a mixed α and β structure, with two α-helices sandwiching antiparallel 3 stranded β-sheet strands. The C-terminal helix of NTL9 forms part of the connection with the C-terminal domain, but there are no contacts between the N and C-terminal domains (Figure 1A–C).^44^

**Figure 1:**
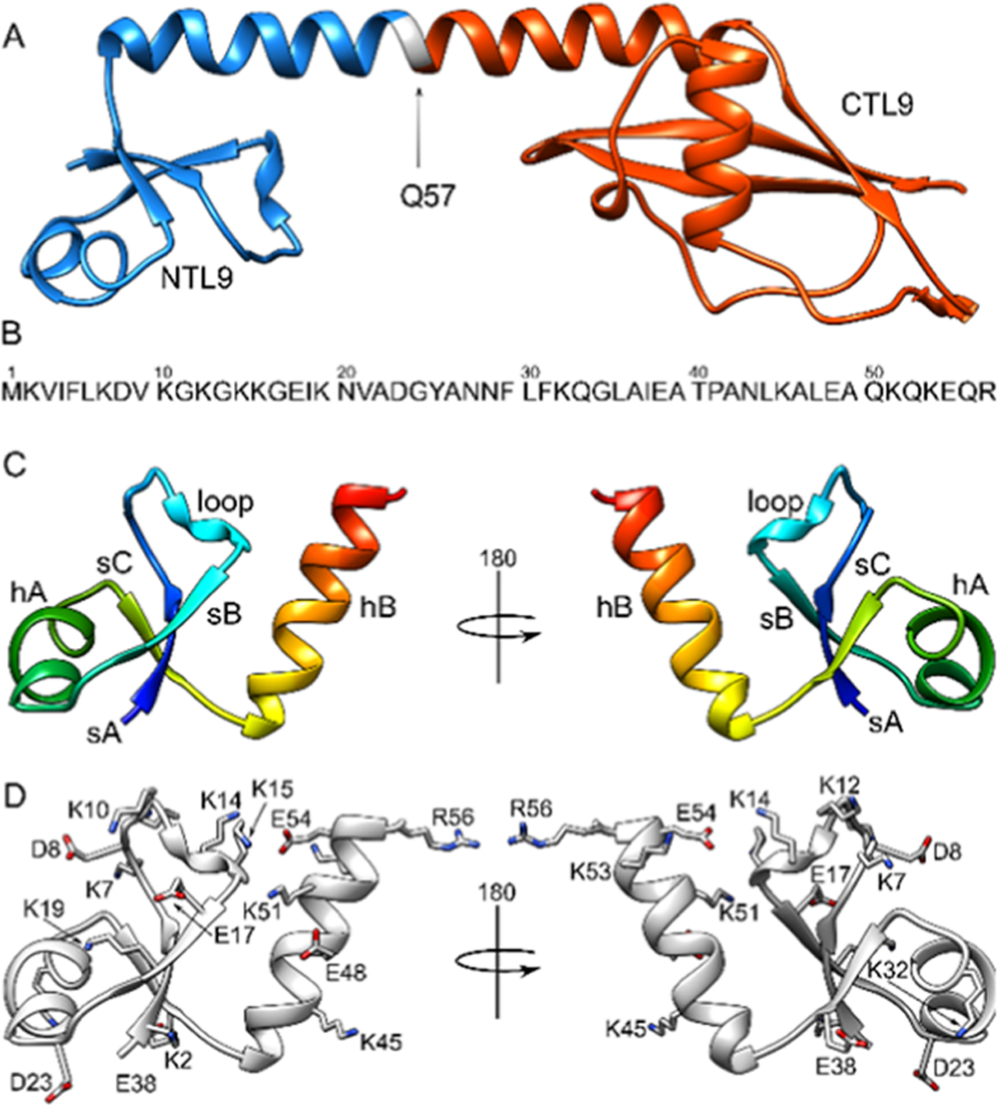
(A) Structure of the ribosomal L9 protein from *Geobacillus stearothermophilus* with the N-terminal construct (NTL9) colored in blue and the C-terminal construct (CTL9) colored in red. The Q57 residue, which is not included in either construct, is labeled. (B) NTL9 sequence. (C) NTL9 ribbon structure is colored in rainbow from N- to C-terminus, with the α-helices (hX) and β-strands (sY) labeled, as well as a loop that appears to show structure. (D) Location of the charged residues in NTL9. A ribbon structure is shown in gray with charged residues (aspartic acid (D), glutamic acid (E), lysine (K), and arginine (R)) in stick format, with nitrogens and oxygens in blue and red, respectively. Based on structure PDB ID: 1DIV.^44^

In this study, we combine native IM-MS and *in silico* unfolding of NTL9 to create an *in vacuo* model of unfolding. Upward of 100 repeats of an unbiased *in silico* method of thermal unfolding, using the approach described in Popa *et al*.,^40^ were performed. The unfolded models match the *in-solution* unfolding and are in line with *in vacuo* data. While the models of unfolding are in good accordance, there are discrepancies between experimental and theoretical CCS values of the final models. Analysis of the deviations provides clues to important factors which may affect the analysis and comparison of *in-solution* and *in* vacuo unfolding.

## Methods

### Sample Preparation

NTL9 was produced and purified as described previously.^52^ Lyophilized NTL9 was dissolved in 100 mM ammonium acetate pH 7.5 to 50 μM and frozen at −20 °C. On the day of data collection, the sample was desalted by buffer exchange using Amicon ultra centrifugal filtration units (Merck Millipore, U.K.), 6 times centrifuged at 14.0E3 g for 15 min at 4 °C using a Heraeus Fresco 17 centrifuge. The concentration was analyzed by Qubit assay (Thermo Fisher Scientific, U.K.).

### Data Collection

Samples were directly infused into the mass spectrometer using nESI from gold-coated capillaries prepared in-house using a Flaming Brown P97 needle puller (Sutter Instruments Co) and a Q150R S sputter coater (Quorum Tech, U.K.). Single-stage IM data was collected on a Synapt G1 (Waters Corp, U.K.) using the parameters presented in Table S1. CCS measuring by TWIMS (^TW^CCS_N2→He2_, following notation described in Gabelica *et al*.^56^) calibration was performed using melittin (Sigma, U.K.), human insulin (Sigma, U.K.), ubiquitin (Sigma, U.K.), equine cytochrome C (Merck Millipore, U.K.), and β-Lactoglobulin (Sigma, U.K). (Figure S2).

### Simulations

The NTL9 structural model was created from residues 1–56 of the full L9 protein (PDB ID: 1DIV).^44^ MD simulations were performed using Gromacs v2018.4. The simulation pipeline (see Figure S1) is as follows: the initial structure is checked for completeness, *i.e.*, all residues contain all atoms. The version of the mobile proton algorithm^40,41^ (MPA) used during simulations requires nonchargeable side-chain residues at the N- and C-termini, so a C-terminal glycine residue was added *via* Modeller (v9.23); however, both the N- and C-termini remain chargeable. The Avidin model was supplied by the Konermann group and is based on PDB ID: 5IRU.^57^ Initial protonated and deprotonated topology files were created using *pdb2gmx* with the OPLSAA force field;^58^ the input values for the state of lysine, arginine, glutamate, aspartate, histidine, and termini were created using a python script (available at https://github.com/ThalassinosLab/charge_site_calculator). A charge library was created by copying the residue information from both the protonated and deprotonated topology files. A GROMACS and structure file was then created for the specific charge state. The MPA was used to distribute the protons across the structure, before equilibration. After equilibration and minimization (Tables S2–S4), a 20 ps simulation at the set temperature was run, after which the protons were rearranged. This cycle continued until the full time period had elapsed, which was either 4 ns at a set temperature (for thermal unfolding) or 100 ns (for coulombic unfolding) (Figure S1).

### Data Analysis

IM-MS spectra were analyzed using MassLynx v4.1 (Waters Corp, U.K.) and DriftScope v2.1 (Waters Corp, U.K.). CCS calculations were performed using the spreadsheet available from http://www.homepages.ucl.ac.uk/~ucbtkth/resources.html, which uses the method of Thalassinos *et al*.^59,60^ and ^TW^CCS_N2→He2_ values derived experimentally from first principles by Bush *et al*.^61^ CCS distribution (CCSD) metrics intensity weight mean (IWM) and intensity weight standard deviation (IWSD) were calculated using the following equation
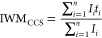
where *I* is the intensity for each arrival time, *t* is the arrival time, and *n* is the number of data points in the arrival time axis.^62^

For the simulations, the theoretical CCS (^TH^CCS) of each frame was calculated every 2 ps for the heating simulations and every 10 ps for the charging simulations using the IMPACT^63^ pseudo-trajectory method, and the final structure was calculated using Collidoscope.^64^ Salt-bridge analysis was performed using VMD (v1.9.2)^65^ using a distance cutoff of 4 Å between the oxygen of an acidic residue and the nitrogen of a basic residue to identify salt-bridge pairings. Ensemble cluster analysis was performed using Chimera (v1.1.3).^66^

## Results

### Summary of Structure and Solution Unfolding of NTL9

The folding and unfolding of NTL9 in solution occurs in a two-step process, progressing from a folded globular to a transition state, onward to the unfolded state.^46,47^ During folding, 60–65% of the total native solvent accessible area is buried in the transition state.^45^ The C-terminal helix, hB, does not form any electrostatic salt bridges to the globular structure, although there are potential intra-helical salt bridges. The last few residues of the C-terminal helix are frayed in solution, and the helix likely undergoes additional fraying during thermal denaturation prior to full unfolding of the globular structure. The C-terminal helix is also partially populated in isolation.^48^ Removal of the final 5 residues, _51_KQKEQR_56_, destabilizes the domain.^51^ Residues D8, E17, and D23 (Figure 1D) form interactions that are perturbed during unfolding.^48^ D8 is in a partially ordered loop which includes 5 lysine residues (_7_KDVKGKGKK_16_) and may form electrostatic contacts with several different side chains, E17 contacts the amide group of K14, and D23 forms a strong salt bridge with the N-terminal amino group.^48^ During thermal unfolding, the core of the structure consisting of the first helix and β-sheet likely, comprising the first 39 residues, unfolds after the unfolding/fraying of the C-terminal helix. The first 39 residues of the protein can fold in isolation but are prone to aggregate in solution.^48^

### ^TW^CCS Analysis

**Figure 2:**
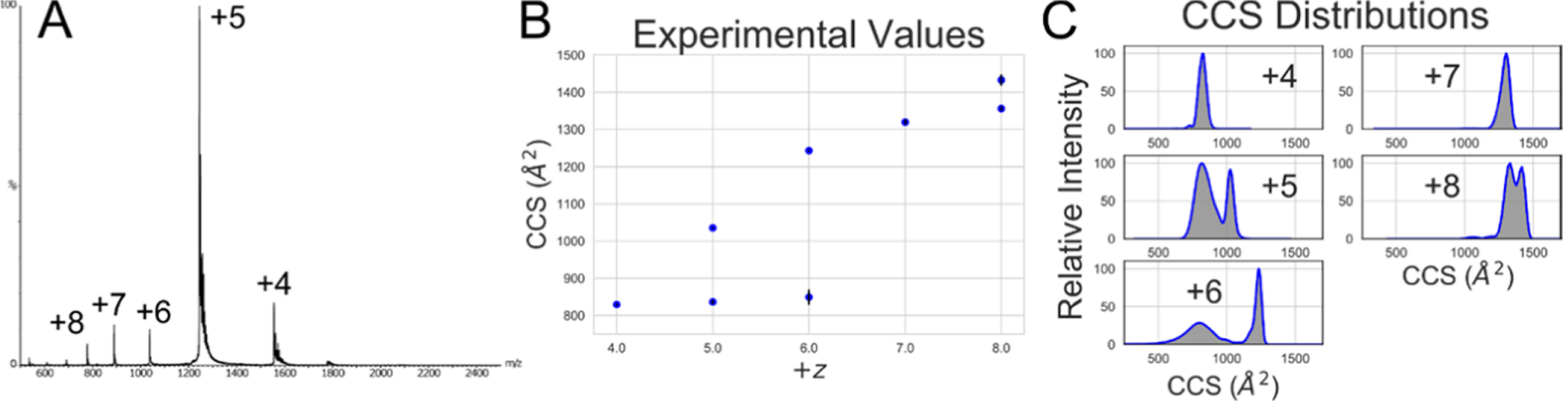
Representative experimental IM-MS data for NTL9. (A) Mass spectrum with charge states labeled; (B) average CCS peak top values; and (C) an example of CCS distributions.

NTL9 displays charge states ranging from +4 to +8, and there appear to be two overlapping charge state distributions (CSDs) (Figure 2A,B). IM-MS analysis shows that charge states from +4 to +6 have a compact form, and states from +5 to +8 have an extended form. This suggests that NTL9 occupies a compact state of approximately 840 Å^2^, which is retained between +4 to +6, and extended states, which are maintained by intramolecular coulombic repulsion, going from 1030–1430 Å^2^. The compact peaks for +5 and +6 show large FWHMs, suggesting that they contain a multiplicity of conformations.(Table 1)

**Table 1:** Experimental CCS Values

+*z*	^TW^CCS_N2→He2_ (Å^2^)
4	829 ± 3.24
5	837 ± 5.47, 1036 ± 2.15
6	850 ± 20.69, 1243 ± 2.65
7	1319 ± 6.97
8	1356 ± 5.01, 1433 ± 15.12

Activation of the NTL9 charge states leads to the +4 state coming slightly more extended; every other charge state either transitioned into a previously present extended state or did not unfold (Figure S3).

### Thermal Unfolding Simulations

**Figure 3:**
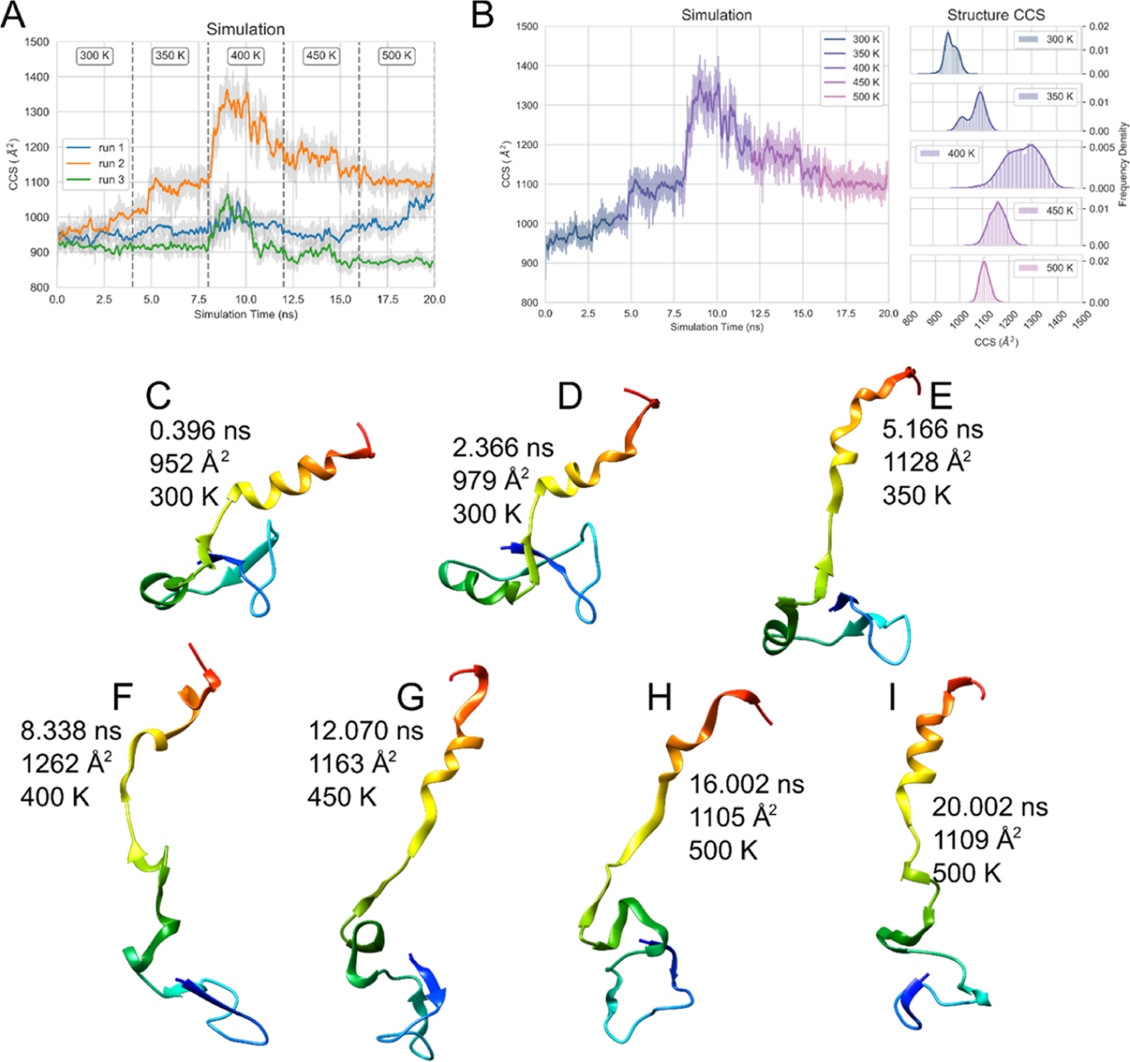
Simulation outcomes of the unfolding of the +5 charge state by heating. (A) Initial triplicate of simulations, with the different temperature transitions marked out with dotted lines. (B) Simulation of the +5 state, which displayed unfolding, with histograms of the states at each temperature shown to the right. The shading represents the unsmoothed data, and the solid line represents smoothed with a mean window of 50, once. (C–I) various unfolded states of the +5 model with the time elapsed, theoretical CCS, and simulation temperature.

Initial thermal unfolding simulations were performed in triplicate on the +5 charge state, as it is experimentally the most intense and is the lowest charge state to display a clear conformational change during collision activation (Figure S3). The thermostat was increased by 50 K over 4 ns for a total simulation time of 20 ns.^40^ One of the triplicates unfolded after approximately 8 ns once the temperature had increased to 400 K (Figure 3A,B); however, it recompacted under increased heating after the initial unfolding event.

The simulation suggests an unfolding pathway where there is C-terminal unfolding, which, for example, can be shown by the formation of a salt bridge between E38 and K2 at later frames in the simulation (Figure S4). Other salt bridges which are diagnostic of particular *in silico* conformations are E48/54 to K51, which are characteristic of the C-terminal helix (hB).

**Figure 4:**
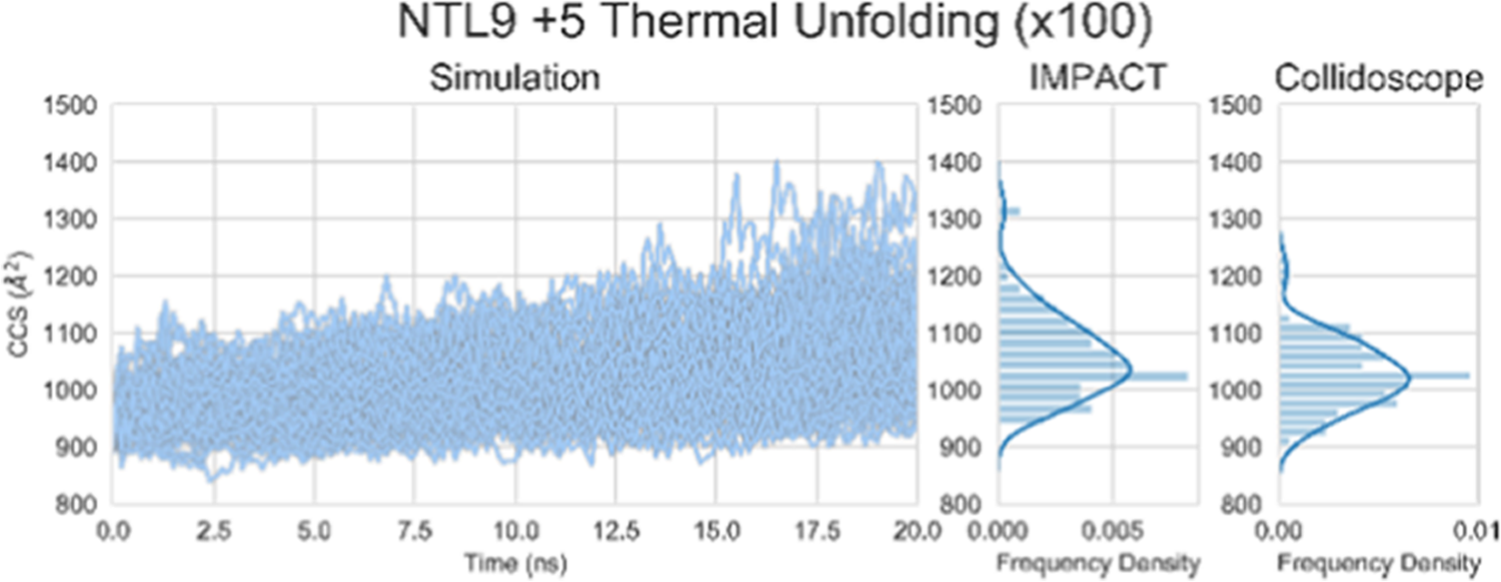
100 replicates of the +5 *in silico* unfolding, showing the trace of the CCS of each frame as calculated by IMPACT, and then histograms of the final states calculated by IMPACT and Collidoscope.

The *in silico* thermal unfolding simulations of the +5 charge state were repeated 100 times (Figure 4). Only 24 outcome structures had ^TH^CCS values >1100 Å^2^ (Table S5), and cluster analysis suggests that 80% remain compact (Figure S6A,B Tables S5, and S7). Inspection of unique structures (structures that did not fall into a cluster ensemble) shows that only 13 structures display dissociation of hB from the protein core. This data suggests low reproducibility of *in silico* thermal unfolding. In many of the final states, structural rearrangement occurs to create a “flattened” structure, where there is a rearrangement of hA, sA, and sB, creating a set of interactions between a series of charged residues, including D8, K12, K14, K15, D48, D54, and R56.

The question of whether the low variability of unfolded outcomes was due to the system or the method was explored. NTL9 is from a thermophilic organism, and the domain is thermally stable, with a melting temperature of 78 °C at pH 5.4 in solution^44^ and therefore *in silico* thermal unfolding may not be appropriate. We attempted to replicate some of the observations from the original publication, which showcased *in silico* thermal of unfolding using the MPA. In the original publication, the homotetramer transthyretin (TTR) unfolding was simulated, and the authors were able to demonstrate charge-mediated subunit ejection, which is consistent with the observation of subunit dissociation and charge stripping observed in IM-MS studies,^8,67−69^ within 20 ns of simulations. Consequently, we explored the thermal *in silico* unfolding of a tetrameric protein of similar mass and structure, Avidin. None of the 100 × 20 ns simulations displayed subunit ejection; however, a partial unfolding of subunits was observed, presumably as a precursor to ejection (Figure S5 Tables S6, and S7). This suggests that the original MPA workflow does not reproduce thermal unfolding *in silico* in a reliable manner.

### Coulombic Unfolding Simulations

**Figure 5:**
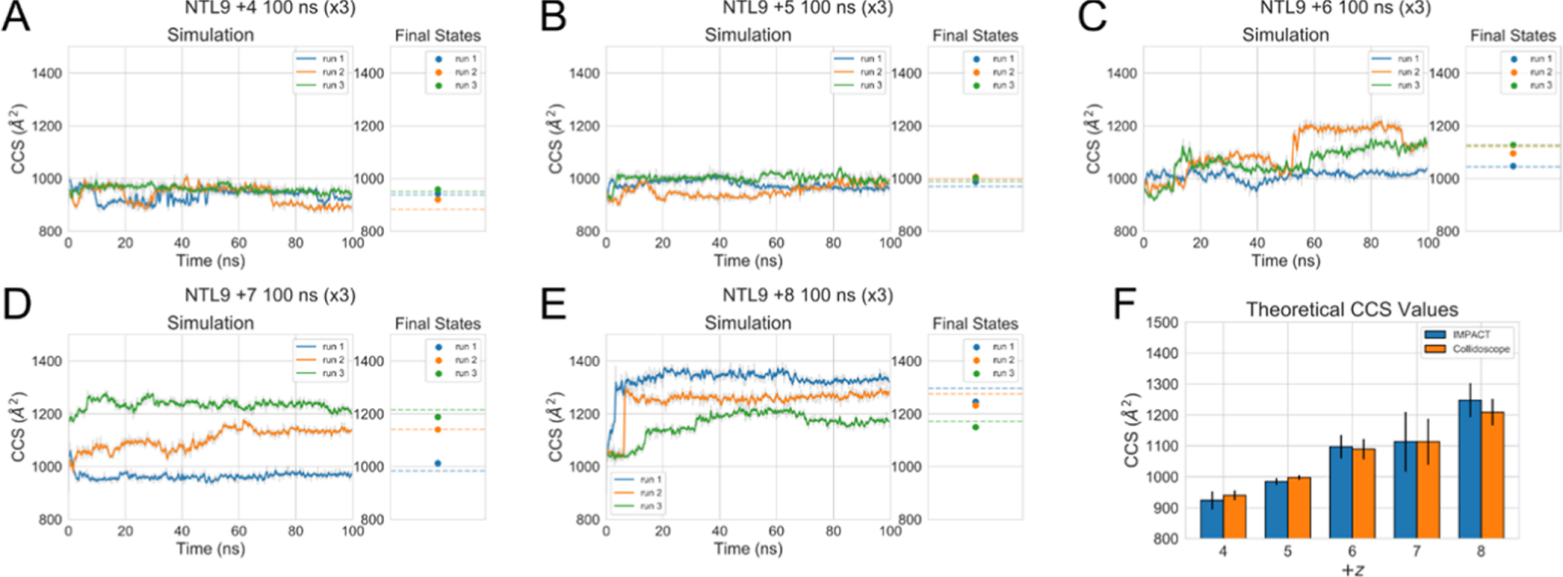
Theoretical CCS values of initial simulations, beginning from the same set with IMPACT analysis of full simulations in solid lines, with a separate plot showing the IMPACT value of the final structure shown as a dotted line and Collidoscope value as dots. Triplicate simulations of (A) +4, (B) +5, (C) +6, (D) +7, and (E) +8 charge state. The gray shading represents the unsmoothed data, and the solid line represents smoothed with a mean window of 5, once. (F) Mean ^TH^CCS values of the final states of the simulations calculated by IMPACT and Collidoscope.

Since the *in silico* thermal unfolding showed low reproducibility, we next investigated whether increasing the charging of the protein would lead to a better match with the *in vacuo* experimental observations. Simulations were performed for the +4 to +8 charge states at a steady temperature of 300 K, for 100 ns. As the charge increases, the theoretical CCS (^TH^CCS) of the final state increases in line with the native IM-MS data (Figures 5 and S7–S11). While the ^TH^CCS of the *in silico* model increases, not all runs lead to an extended state, for instance, for the +7 state, run 1 retains a compact structure (Figures 5 and S9A). For each run, the root-mean-square deviation of the Cα atoms between the frame and the final state decreased and became mostly stable (Figure S12).

**Figure 6:**
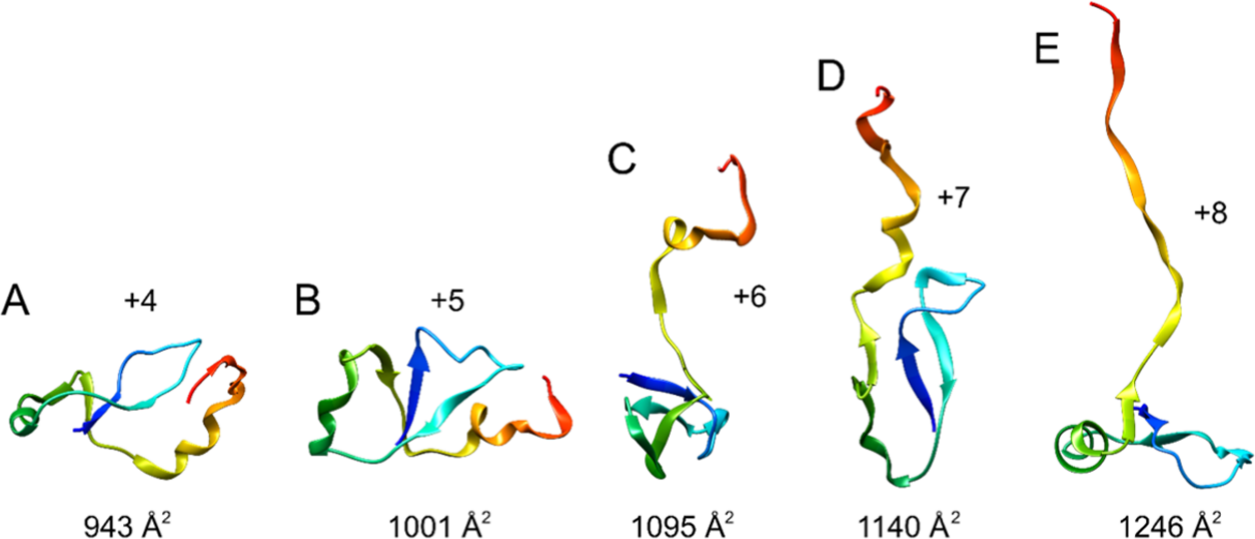
Selected final structures for the charging simulations after 100 ns for charges (A) +4, (B) +5, (C) +6, (D) +7, and (E) +8, with the theoretical CCS value calculated by Collidoscope.

The coulombic simulations show a consistent evolution of *in vacuo* structures (Figure 6), with +4 and +5 forming compact structures and +6 to +8 forming more unfolded structures, with the thermally unfolded +5 and coulombically unfolded +8 *in silico* models having comparable structures.

To properly compare the *in silico* and *in vacuo* data, each of the coulombic simulations was repeated 100x, to create a theoretical CCS distribution (^TH^CCSD) from a kernel density estimation of the final *in silico* models, which could then be compared to the experimental data. Replication instead of increasing the length of the simulations was chosen, as it was clear that 100 ns was a long-enough time period for a conformational sampling of an extended state to occur from the initial triplicates.

**Figure 7:**
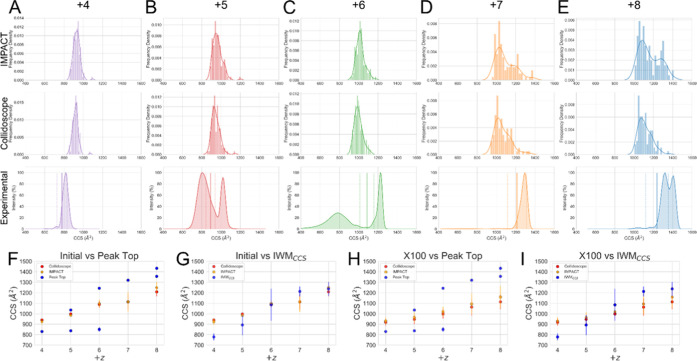
Comparison of simulated and experimental results. (A–E) Results of 100 replicates of the stable temperature simulations showing histograms of the final states calculated by IMPACT and Collidoscope and experimental CCS distributions, with the IWM_CCS_ denoted by the dotted line, and the standard deviation shown by the faint dotted lines for (A) +4, (B) +5, (C) +6, (D) +7, and (E) +8 charge states. (F–I) Comparison of theoretical and experimental values. Mean ^TH^CCS value from initial triplicate simulation final states plotted against (A) experimental peak top CCS values and (B) IWM_CCS_. Mean ^TH^CCS value from X100 simulation replicate final states plotted against (C) experimental peak top CCS values and (D) IWM_CCS_.

A comparison of the ^TH^CCSDs (Figure 7) shows that increasing the replicates creates a better likelihood of a model matching the *in vacuo* data. Interestingly, all of the ^TH^CCSDs appear to show some degree of multimodal behavior. Ensemble cluster analysis of the 100 final structures of each simulated charge state support this (Figure S18). Comparison of the theoretical and experimental CCSDs shows that while a high replication number is not able to reproduce something which exactly matches the experimental distribution, an overlap exists between the ^TH^CCS of some models and experimental values, meaning a subset of models reproduce the experimental data.

## Discussion

Creating robust pipelines for modeling *in vacuo* unfolding *in silico* would be highly advantageous for understanding protein behavior during IM-MS analysis. It would also be advantageous to be able to objectively evaluate if the unfolding observed *in vacuo* is relevant to *in-solution* behavior. This would help CA become a more informative structural biology technique: in much the same vein as how the demonstrations that soft ionization retains the solution-like structure and behavior^70^ facilitated the use of mass spectrometry a valuable tool for structural biologists.

NTL9, a well-studied system in-solution,^45−51^ appears to adopt a mixture of compact and extended states during ionization, which are observable *in vacuo* (Figure 2B,C). Trying to reproduce these states in an unbiased manner *in silico*, and hence create a model of unfolding, to allow comparison of *in-solution* and *in vacuo* data has had variable success. Both thermal and coulombic unfolding *in silico* matched the expected *in-solution* model: release of the C-terminal α-helix (hB), with dissociation of the central β-sheet and the first α-helix (hA). Replications of the *in silico* thermal unfolding did not show good reproducibility, and the original method for thermal unfolding^40,41^ produced unfolded structures only 13/100 times. This is most likely due to the lack of conformational sampling present in unbiased simulations and was not limited to the thermostable system NTL9.^71−73^ While the test system previously used in Popa *et al*., to demonstrate the utility of the MPA workflow was transthyretin (TTR),^40^ another well-studied homotetrameric system, Avidin, was used here. Avidin has a slightly higher intact mass than TTR, 64.0 kDa compared to 55.0 kDa; the structure used in our studies is truncated at the C-terminus, making it 54.8 kDa and similar in mass and size to TTR, meaning they should have a similar internal temperature when heated.^57^ In the original *in silico* studies, charge-mediated subunit ejection was observed for TTR; however, Avidin displayed no charge-mediated subunit ejection on the timescale of our simulations. The TTR structure used previously (3GRG) is in fact a mutant known as M-TTR (F87M/L110M).^74^ M-TTR shows a reduced self-association constant due to the substitution of F87 and L110 with the bulkier M residues at subunit interfaces,^74,75^ which may have affected the outcome of the simulations. The lack of subunit ejection observed in Avidin suggests that the thermal unfolding workflow has limited conformational sampling, which may be exacerbated by the proton hopping. Energy minimization studies^64,76^ show that 10^6^–10^7^ proton rearrangements need to be performed to reach an energy minima, which adds an extra layer of complexity outside of standard structural dynamics. While in this study, we opted for more replicates of shorter simulations, fewer numbers of longer, biased simulations may be required. Longer simulations may also allow the creation of more accurate theoretical CCSDs: while experimental CCSDs are a product of gas-phase conformations which do not interconvert on the timescales of drift separation, they are the result of dynamics of timescales exceeding the simulation time.

Comparison of ^TW^CCS and ^TH^CCS values is an important part of bridging the gap between *in vacuo* and *in silico* studies. Here, we have compared several metrics which are used experimentally for IM-MS analysis: the peak top value and the intensity weighted mean (IWM) (Figure 7G,I). Comparison of the peak top values to the mean ^TH^CCS values is poor (Figure 7F,H) due to the fact that the average is unable to capture distinct populations. Using a method like IWM_CCS_ gives better overlap as it better captures the weighting of multi-conformer ensembles.

From the simulations, high charging, *i.e.*, +7 and +8, produce highly unfolded *in silico* structures; however, ^TW^CCS values suggest that *in vacuo*, more compact conformers are favored. The discrepancy between the ^TW^CCS and ^TH^CCS may be due to the difficulty of calculating ^TH^CCS of linear ions, as described by Kulesza *et al*.,^77^ which is highlighted in our study by the differences between the ^TH^CCS values calculated by IMPACT and Collidoscope for the same extended structures of the +7 and+8 charge states (Figures 5E and S18G–J). It may also be a function of ^TW^CCS calibrant class: to get an accurate TWIMS CCS calibration, molecules of the same class as the experimental molecule must be used, *i.e.*, native protein calibrants for globular proteins and denatured proteins for disordered or denatured proteins. The *in silico* structures suggest that the higher charge states of NTL9 have both ordered and disordered structural regions, meaning that neither calibrant class would be fully comparable. Other possible avenues for future exploration to close the gap between the ^TH^CCS and ^TW^CCS include using different force fields during *in silico* model creation, as studies have shown that certain force fields, which are designed to replicate *in-solution* behavior, commonly produce models which favor either compaction or extension compared to experimentally derived CCS values.^78^ Furthermore, different methods to produce simulated ions could also be employed, as the complexity of comparing the molecular dynamics to experimental data is compounded by the differing behavior of folded and unfolded protein ions during desolvation. While folded proteins are regarded as ionizing *via* the charged residue model (CRM),^79−83^ unfolded proteins are believed to ionize *via* the chain ejection model (CEM).^84,85^ The two models could imply differing unfolding mechanisms, which the MPA may not be able to replicate fully. Desolvation simulations function by steadily removing solvent from a charged droplet containing the protein structure to create a gas-phase ion.^31,32,86−90^ While the simulation of the droplet itself is more computationally expensive, it may produce a more accurate initial structure for further simulation.

By combining *in vacuo*, *in silico* and *in-solution* data, we have shown it is possible to create coherent models of unfolding that link gas-phase and solution-phase behaviors. This approach shows promise and importantly, highlights multiple experimental and theoretical avenues to explore to further improve the methodology. We believe that the data and the analysis presented here both illustrate the power of the hybrid experimental computational approach and point the way for future developments.

## Supporting Information Available

*in silico* pipeline, *in silico* parameter tables, experimental parameter tables, CCS calibration charts, salt-bridge analysis, triplicate final structures of coulombic simulations, RMSD charts, full tables of *in silico* CCS values for 100× coulombic and thermal simulations, and ensemble analysis for simulations (PDF)

## Abbreviations Used

CA: collision activation
CCS: collision cross-section
CD: circular dichroism
CIU: collision induced unfolding
CTL9: C-terminal construct of the L9 protein
EPR: electron paramagnetic resonance
IM: ion mobility
IM-MS: ion mobility mass spectrometry
IWM: intensity weighted mean
IWSD: intensity weighted standard deviation
MD: molecular dynamics
MPA: mobility proton algorithm
MS: mass-spectrometry
NMR: nuclear magnetic resonance
NTL9: N-terminal construct of the L9 protein
^TH^CCS: theoretical collision cross section
TTR: transthyretin
^TW^CCS: experimental collision cross section
