# Supplementary material for: Linking Gas-Phase and Solution-Phase Protein Unfolding via Mobile Proton Simulations
Source: Anal Chem. 2022 Nov 9;94(46):16113–21. doi: 10.1021/acs.analchem.2c03352 (PMC9685592; doi:10.1021/acs.analchem.2c03352)
Supplement: Supplementary file 1 — ac2c03352_si_001.pdf [file ac2c03352_si_001.pdf]

Linking Gas-Phase and Solution-Phase Protein Unfolding Via Mobile Proton Simulations: Supplementary Information

Charles Eldrid[1,2], Tristan Cragnolini[3], Aisha Ben-Younis[2], Junjie Zou[4], Daniel Raleigh[2,4], Konstantinos Thalassinos*[2,3]

1. School of Biological Sciences, University of Southampton, Southampton, SO16 1BJ, UK 2. Institute of Structural and Molecular Biology, Division of Bioscience, University College London, London, WC1E 6BT, UK 3. Institute of Structural and Molecular Biology, Birkbeck College, University of London, London, WC1E 7HX, UK 4. Department of Chemistry, Stony Brook University, 100 Nicolls Rd, NY 11794, USA

Methods and Experimental Parameters

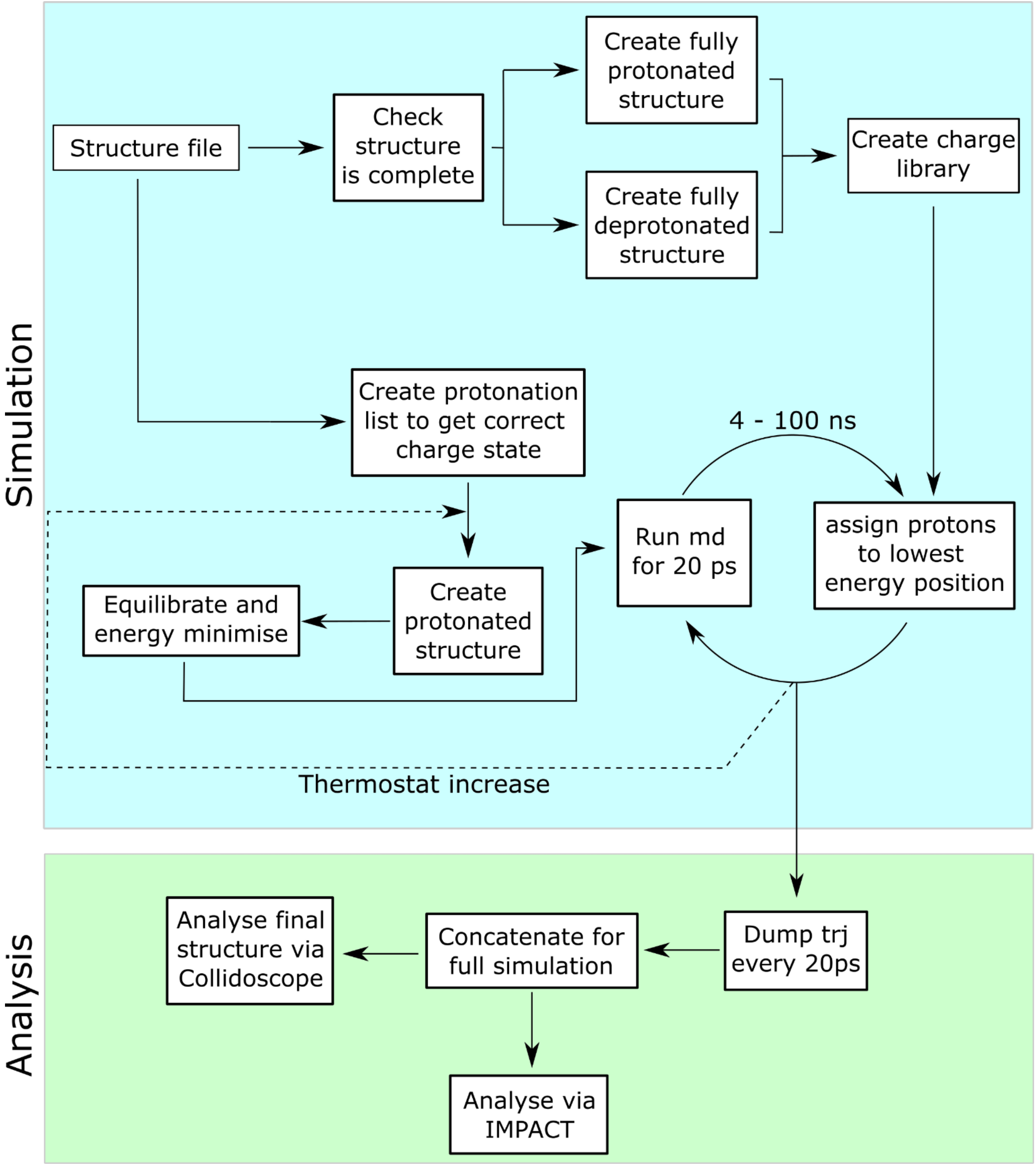

Figure S1: Pipeline for execution and analysis of mobile proton simulations, following the workflow set out by the Konnerman group.

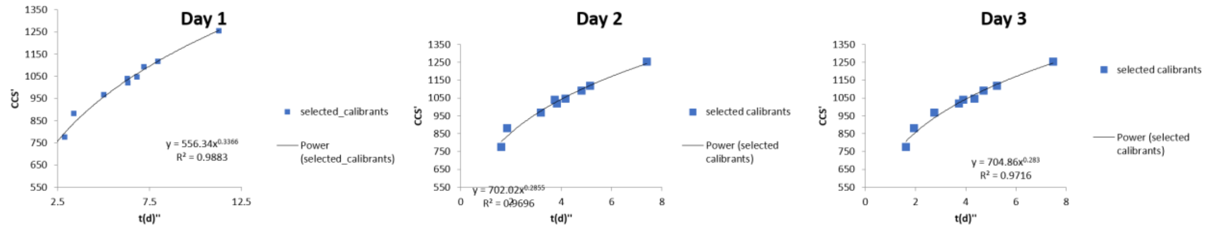

Figure S2 ^{TW}CCS calibration graphs for NTL9 IM-MS data on the Synapt G1

Parameter	Value	Mobility Parameter	Value
Capillary (kV)	1.1	Source wave velocity (m/s)	300
Sampling cone (V)	30	Source wave height (V)	0.2
Extraction cone (V)	0.5	Trap wave velocity (m/s)	300
Backing pressure (mbar)	3.20	Trap wave height (V)	0.2
Trap CE (eV)	5	IMS wave velocity (m/s)	200
Transfer CE (eV)	3	IMS wave height (V)	4.5
Bias	6	Transfer wave velocity (m/s)	200
Mass range (m/z)	1000 - 7000	Transfer wave height (V)	4.5

Table S1 Experimental acquisition parameters for IM-MS data on Synapt G1

Parameter	Value	Parameter	Value
Constraints	All-bonds	rcoulomb	333.3
Integrator	md	rvdw	333.3
Dt	0.002	pbc	Xyz
Nsteps	50,000	Gen-seed	109
Nstxout-compressed	1000	Gen_vel	Yes
Nstlist	50	Gen_temp	1
Ns_type	Simple	Tcoupl	Nose-hoover
Cutoff-scheme	Verlet	Ref_t	1
Comm_mode	Linear	Tau_t	0.1
Coulombtype	Cut-off	Tc-grps	System
Vdwtype	Cut-off	nsttcouple	1
rlist	333.3	Pcoupl	NO

Table S2 Gromacs parameters for equilibration

Parameter	Value	Parameter	Value
Integrator	Steep	Rlist	333.3
Emtol	1000.0	Coulombtype	Cut-off
Emstep	0.01	Rcoulomb	333.3
Nsteps	50,000	Vdwtype	Cut-off
Nstxout-compressed	1000	Pbc	xyz
Cutoff-scheme	Verlet	nstlist	1
Ns_type	grid		

Table S3 Table of Gromacs parameters for energy minimisation

Parameter	Value	Parameter	Value
Constraints	All-bonds	rcoulomb	333.3
Integrator	md	rvdw	333.3
Dt	0.002	pbc	Xyz
Nsteps	10,000	Gen-seed	109
Nstxout-compressed	1000	Gen_vel	Yes
Nstlist	50	Gen_temp	300-500
Ns_type	Simple	Tcoupl	Nose-hoover
Cutoff-scheme	Verlet	Ref_t	300-500
Comm_mode	Linear	Tau_t	0.1
Coulombtype	Cut-off	Tc-grps	System
Vdwtype	Cut-off	nsttcouple	1
rlist	333.3	Pcoupl	NO

Table S4 Table of gromacs parameters for mdrun

Figures and Data Tables

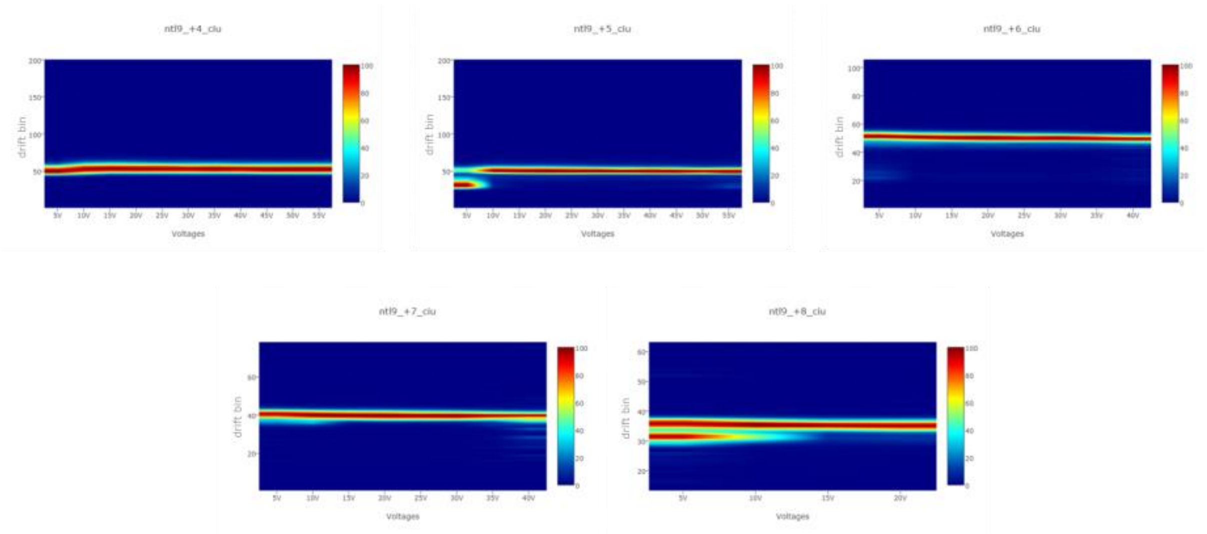

Figure S3 Collision activation fingerprints of NTL9 charge states, without CCS calibration, showing drift bin on the y-axis, and collision voltage on the x-axis, with the intensity scale on the right

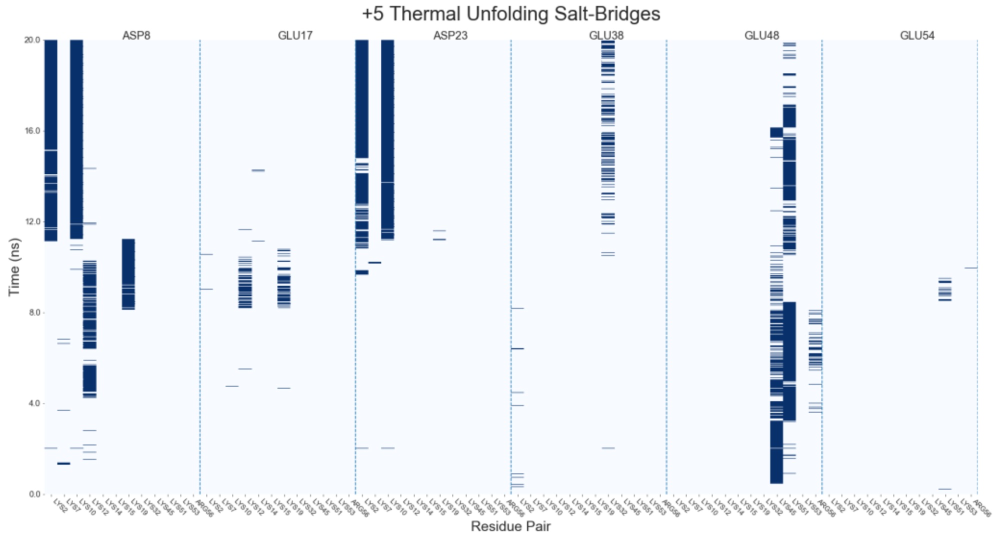

Figure S4 Salt-bridge analysis for run 2 thermal unfolding simulation of the +5 ion. With theoretical acidic/basic residue pairs on the x-axis, and simulation time in ns on the x-axis, with a dark = true, light= false binary for whether or not a salt-bridge is present. The acidic residues are, in order: ASP8, GLU17, ASP23, GLU38, GLU48, GLU54. The basic residues are, in order: LYS2, LYS7, LYS10, LYS12, LYS14, LYS15, LYS19, LYS32, LYS45, LYS51, LYS 53, ARG56.

Run	Impact	Collidoscope	Run	Impact	Collidoscope
1	1086.027	1024.84	51	1105.177	1057.58
2	1051.164	1014.64	52	1043.174	1026.71
3	1056.375	1060.9	53	969.6332	929.779
4	1061.272	1072.23	54	1030.27	956.258
5	1039.268	993.528	55	1159.477	1105.63
6	985.2984	979.82	56	958.5377	924.317
7	1115.809	1083.88	57	1167.522	1104.39
8	967.514	936.27	58	978.3239	979.543
9	1086.967	1027.31	59	1029.304	1077.88
10	988.9691	942.002	60	1044.192	1027.38
11	1127.855	1089.05	61	1135.57	1086.21
12	1107.422	1107.15	62	1014.222	1007.17
13	1067.236	982.789	63	1143.081	1071.56
14	1092.294	1028.71	64	952.4932	932.077
15	1022.122	1009.28	65	1065.648	1009.83
16	1110.179	1116.78	66	1023.887	1062.98
17	1119.558	1051.88	67	1032.152	1009.31
18	938.2766	958.101	68	1032.089	1003.28
19	1054.769	1031.87	69	1087.476	1037.77
20	1148.696	1089.54	70	1043.83	986.373
21	940.2922	901.573	71	966.8861	973.267
22	1032.487	985.731	72	968.8661	921.201
23	1046.891	1021.39	73	1053.541	1009.17
24	1003.252	940.883	74	1197.814	1108.74
25	972.6114	977.564	75	1177.691	1061.36
26	994.6178	1026.1	76	1048.12	1013.25
27	1085.289	1014.34	77	1061.645	993.204
28	1308.518	1190.13	78	1087.755	1054.78
29	1165.133	1093.15	79	1016.572	950.241
30	1017.982	960.202	80	1083.01	1027.13
31	1322.606	1232.91	81	991.3565	966.011
32	1097.553	1092.33	82	955.488	991.154
33	1037.983	1021.23	83	1133.245	1032.28
34	1010.627	1023.58	84	1046.871	1020.03
35	1053.45	1044.13	85	984.1094	975.72
36	1025.141	1053.19	86	994.6191	976.946
37	1021.924	998.371	87	1078.37	1079.43
38	1015.491	1032.72	88	1093.565	1063.59
39	1113.294	1073.65	89	966.5623	954.327
40	1132.299	1047.77	90	1071.962	1029.09
41	1022.095	981.53	91	1085.951	1038.46
42	1017.536	975.699	92	1054.54	1049.52
43	1068.12	1010.25	93	1150.499	1117.73
44	1056.953	1047.21	94	1031.54	1062.01
45	1127.263	1110.61	95	1093.517	1043.13
46	1113.383	1067.97	96	1184.562	1086.45
47	971.6951	988.645	97	1010.019	993.938
48	1017.402	1025.33	98	1012.595	990.688
49	1004.934	1003.66	99	1097.092	1078.45
50	996.3201	976.219	100	1208.048	1208.28

Table S5 Table of IMPACT and Collidoscope values of the final states of the +5 thermal unfolding simulations for NTL9 (x100)

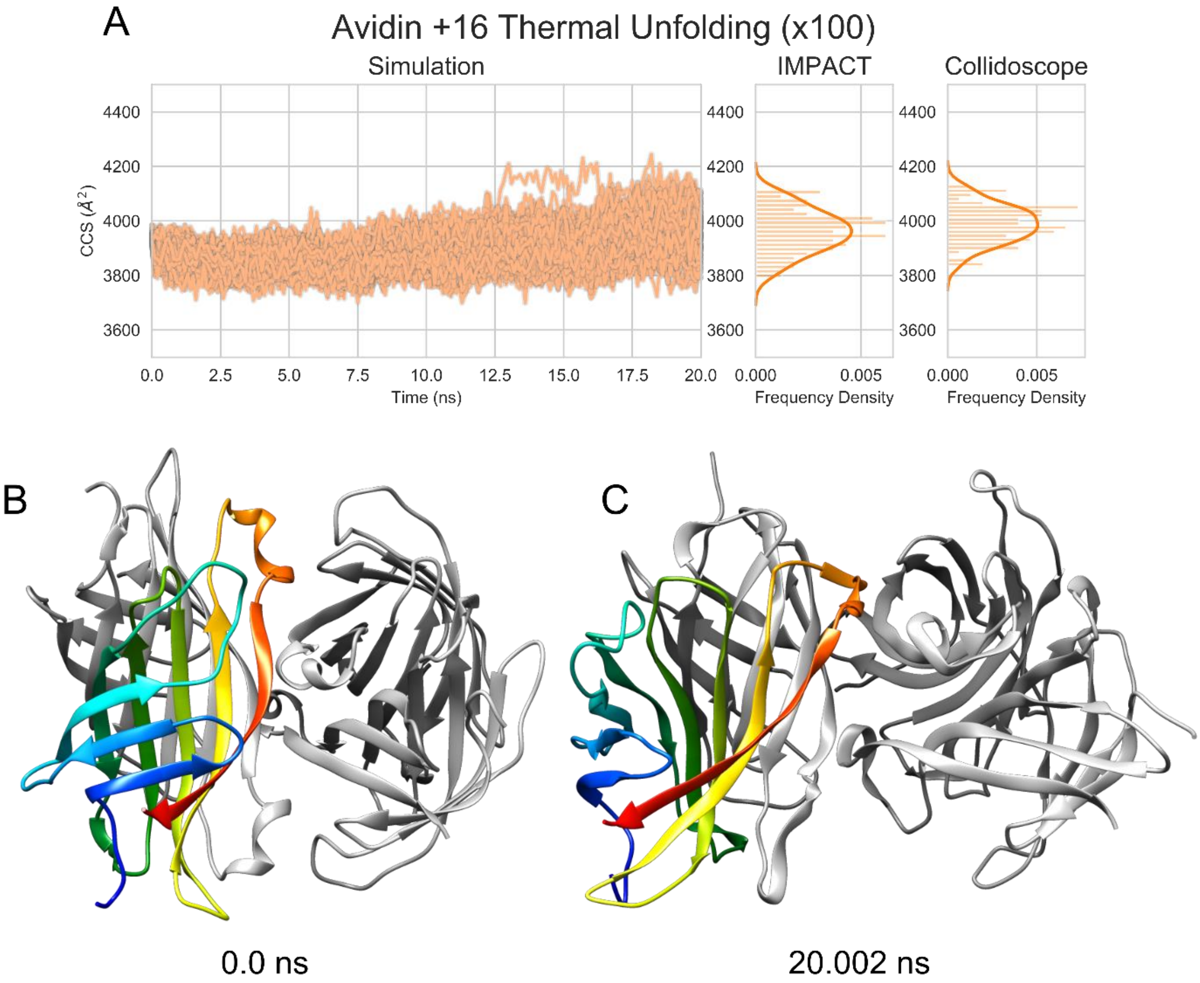

Figure S5: A) 100 replicates of the +16 thermal unfolding simulations, showing the trace of the CCS of each frame as calculated by IMPACT, and then histograms of the final states calculated by IMPACT and Collidoscope. Comparison of the B) beginning frame and C) end frame of run 086 of the thermal unfolding on Avidin +16, displaying partial unfolding of the β structure of a monomer which is highlighted in rainbow

Run	Impact	Collidoscope	Run	Impact	Collidoscope
1	3996.951	3972.93	51	3860.242	3929.97
2	3828.261	3901.64	52	3943.267	3983.38
3	3815.36	3927.33	53	3907.165	3968.92
4	3792.991	3833.08	54	3954.098	4003.14
5	4076.294	4115.64	55	4056.802	4110.34
6	3826.825	3902.32	56	4015.678	4030.77
7	4015.347	4018.17	57	3903.783	3885.91
8	3925.415	3927.53	58	3992.574	3964.2
9	3906.775	3961.18	59	3955.763	3961.82
10	4064.46	4053.03	60	3942.684	4055.41
11	4000.27	4021.19	61	4097.984	4097.69
12	3899.467	3943.41	62	4000.057	4055.97
13	3966.808	4004.11	63	3980.14	4030.19
14	4027.829	4029.11	64	3949.975	4043.89
15	3975.526	4058.06	65	3902.802	3966.49
16	3976.053	4021.37	66	3907.127	3929.69
17	4087.859	4132.93	67	3990.411	3956.09
18	4052.561	4131.34	68	3858.554	3857.05
19	4001.385	4022.76	69	3930.312	3963.23
20	3974.206	3983.62	70	4064.624	4014.14
21	4005.088	4004.31	71	4009.81	3989.96
22	4015.175	4037.12	72	3838.011	3913.82
23	3872.686	3955.2	73	3932.435	3939.85
24	4028.631	4046.16	74	4102.617	4103.77
25	3942.014	3929.98	75	4080.811	4096.4
26	3942.838	3964.72	76	3791.127	3855.32
27	3989.211	4052.27	77	4029.921	4072.35
28	4085.603	4045.77	78	4106.017	4071.99
29	3942.853	3970.63	79	3920.621	3968.89
30	3912.261	4015.6	80	4033.109	4047.64
31	3892.024	3907.06	81	3905.655	3843.56
32	3813.801	3846.89	82	3954.13	3960.62
33	3871.675	3995.99	83	3989.954	3997.39
34	3950.155	3918.73	84	4004.875	4004.11
35	3851.376	3909.2	85	3938.293	3982.45
36	3931	3929.55	86	4113.775	4109
37	3936.262	4036.89	87	3999.096	3975.2
38	3953.078	3978.04	88	4068.98	4032.87
39	3948.985	3968.35	89	3983.651	4025.38
40	4011.866	4047.07	90	4105.08	4112.54
41	3887.859	3897.05	91	3875.748	3901.27
42	3900.299	3900.79	92	3896.536	3925.67
43	3875.917	3943.94	93	3943.17	3980.48
44	4069.071	4040.45	94	3970.393	4002.54
45	4040.468	4029.35	95	3995.404	4017.88
46	4013.877	4047.41	96	3985.466	3972.2
47	3846.358	3908.45	97	4044.563	4057.56
48	3976.028	4080.8	98	3910.036	3945.23
49	3908.838	3951.62	99	3843.586	3921.41
50	3952.658	3986.42	100	4046.389	4001.93

Table S6: Table of IMPACT and Collidoscope values of the final states of the +16 thermal unfolding simulations for Avidin (x100)

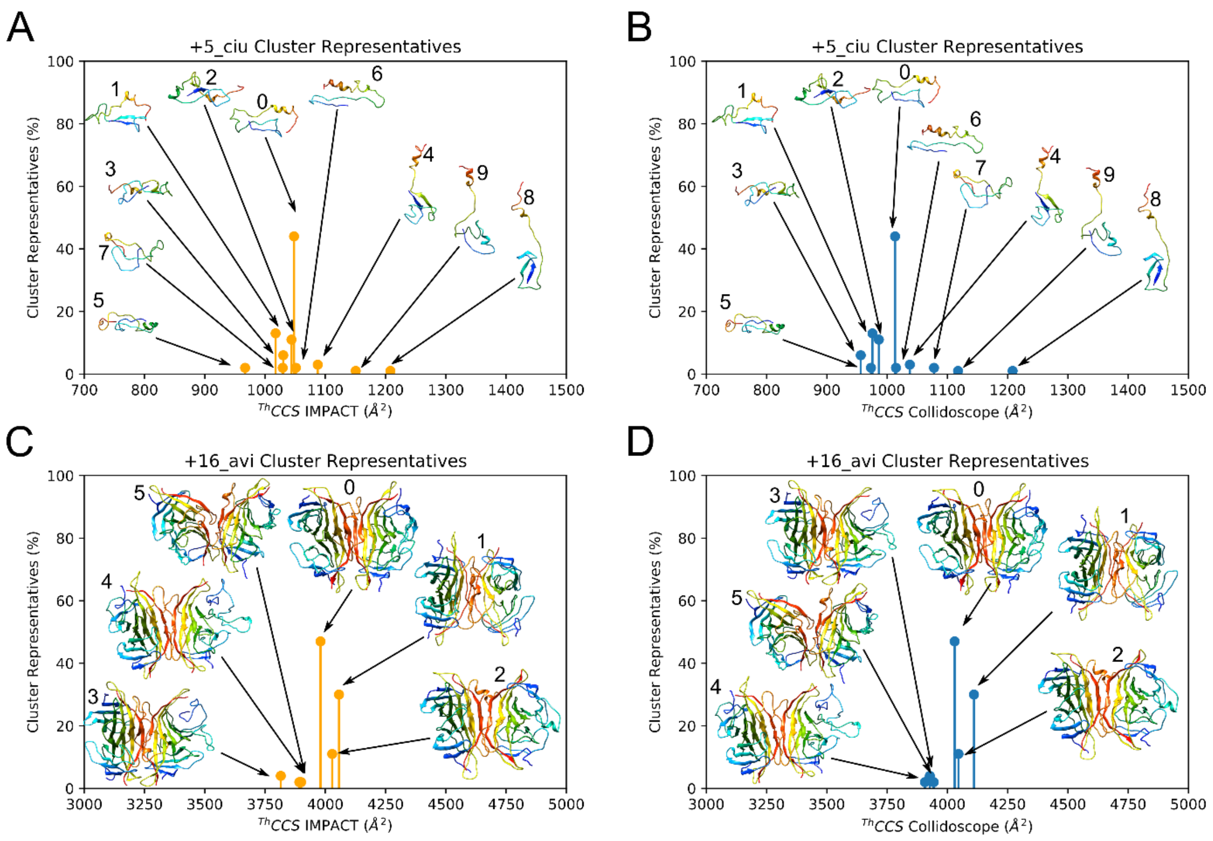

Figure S6: Ensemble cluster analysis of the 100 outcomes of the thermal unfolding simulations, with the THCCS on the x-axis and the percentage of structures represented on the y-axis. Each cluster has the representative structure shown above, with its corresponding rank. These structures represent either the top 10 clusters (NTL9) or ≥95% of outcomes (Avidin). For full data see Table S7. Structures with **A, C)** IMPACT THCCS **B, D)** Collidoscope THCCS **A-B)** +5 NTL9, **C-D)** +16 Avidin simulations.

+z	rank	Percent (%)	Run model	IMPACT (Å²)	Collidoscope (Å²)
+5	0	44	76	1048.12	1013.25
	1	13	42	1017.536	975.699
	2	11	70	1043.83	986.373
	3	6	54	1030.27	956.258
	4	3	69	1087.476	1037.77
	5	2	71	966.8861	973.267
	6	2	2	1051.164	1014.64
	7	2	59	1029.304	1077.88
	8	1	100	1208.048	1208.28
	9	1	93	1150.499	1117.73
+16	0	47	63	3980.14	4030.19
	1	30	55	4056.802	4110.34
	2	11	24	4028.631	4046.16
	3	4	3	3815.36	3927.33
	4	2	31	3892.024	3907.06
	5	2	12	3899.467	3943.41

Table S7 Ensemble cluster analysis outcomes of the 20ns coulombic simulations, showing the charge state, the rank of each cluster, the % of structures that cluster represents, the run number that is representative of the cluster and the THCCS of the structure by IMPACT and Collidoscope.

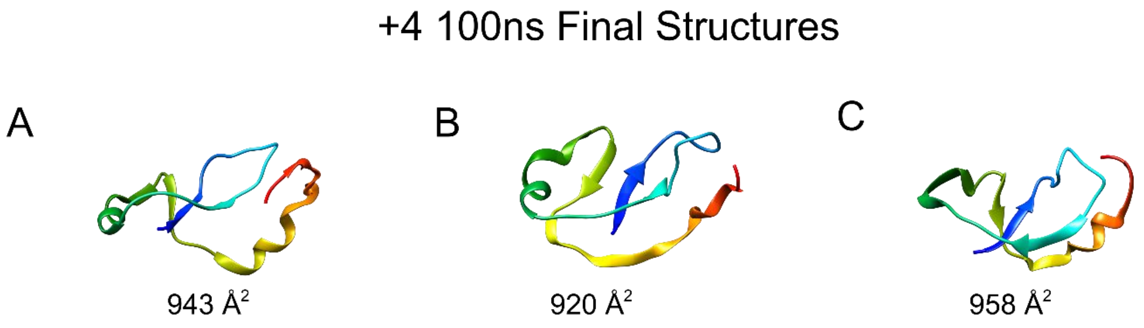

Figure S7 Final structures of the +4 charge state of NTL9 after 100 ns, showing the structure of **A)** run 1, **B)** run 2 and **C)** run 3 with the corresponding CCS calculated from Collidoscope underneath.

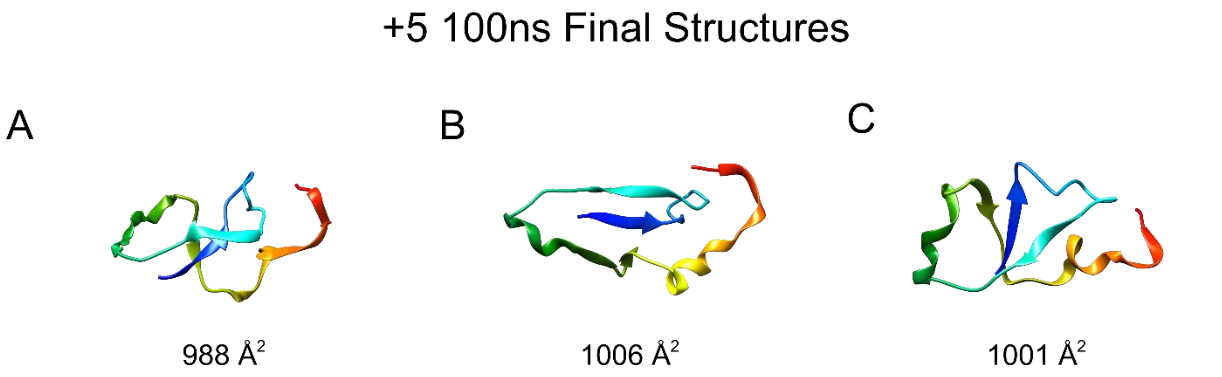

Figure S8 Final structures of the +5 charge state of NTL9 after 100 ns, showing the structure of A) run 1, B) run 2 and C) run 3 with the corresponding CCS calculated from Collidoscope underneath.

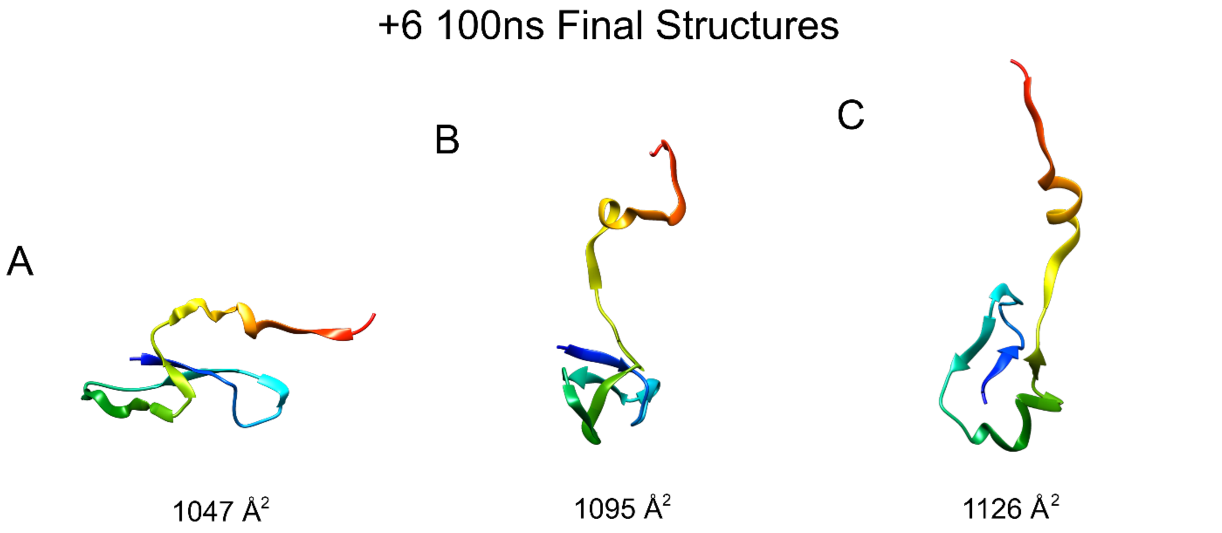

Figure S9 Final structures of the +6 charge state of NTL9 after 100 ns, showing the structure of A) run 1, B) run 2 and C) run 3 with the corresponding CCS calculated from Collidoscope underneath.

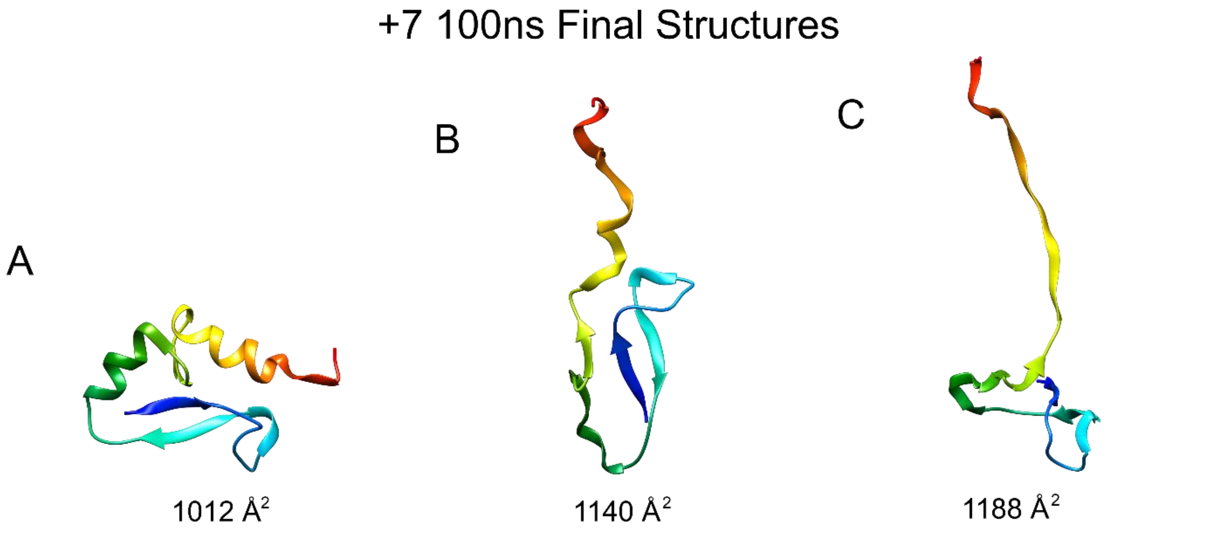

Figure S10 Final structures of the +7 charge state of NTL9 after 100 ns, showing the structure of A) run 1, B) run 2 and C) run 3 with the corresponding CCS calculated from Collidoscope underneath.

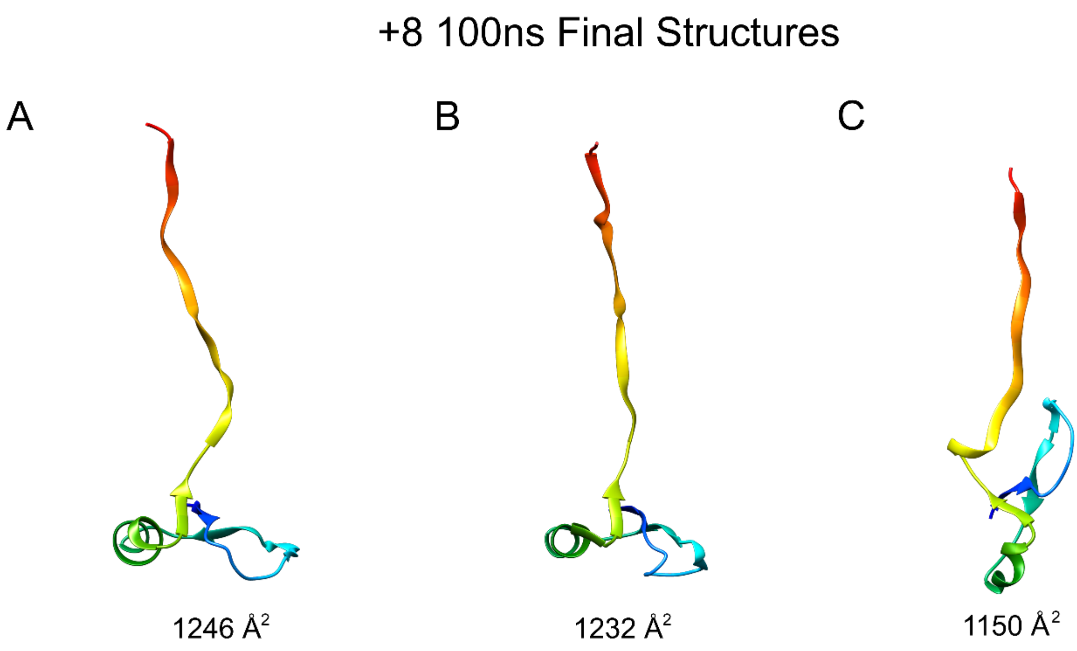

Figure S11 Final structures of the +8 charge state of NTL9 after 100 ns, showing the structure of A) run 1, B) run 2 and C) run 3 with the corresponding CCS calculated from Collidoscope underneath.

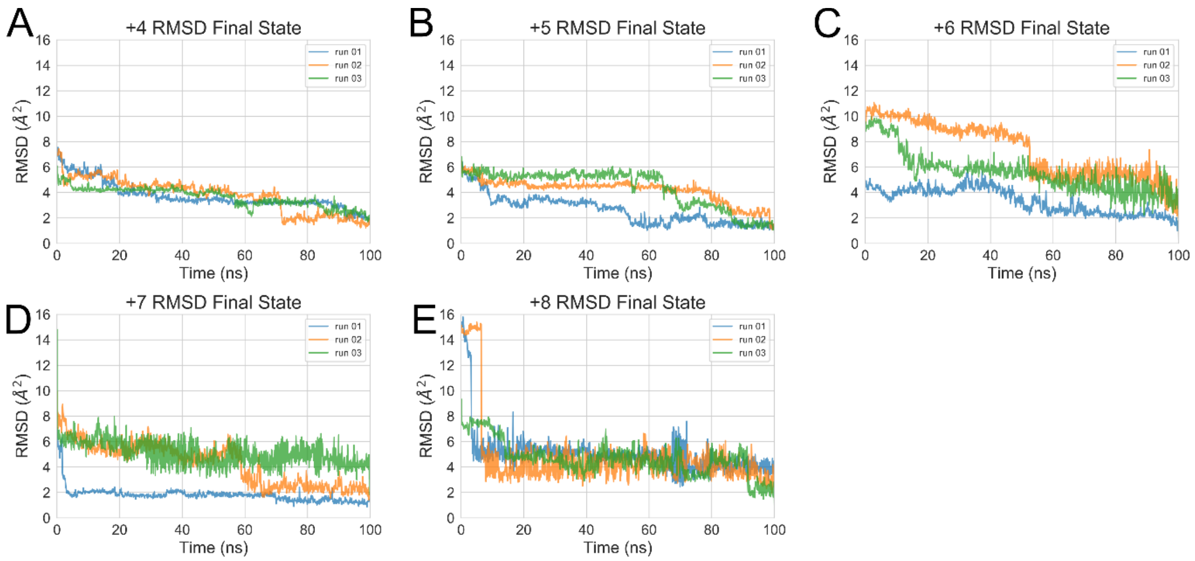

Figure S12 RMSD of the simulation frame compared to the final outcome, showing a tendency for the system to stabilise over 100 ns, for A) +4, B) +5 C) +6 D) +7 and E) +8 charge state.

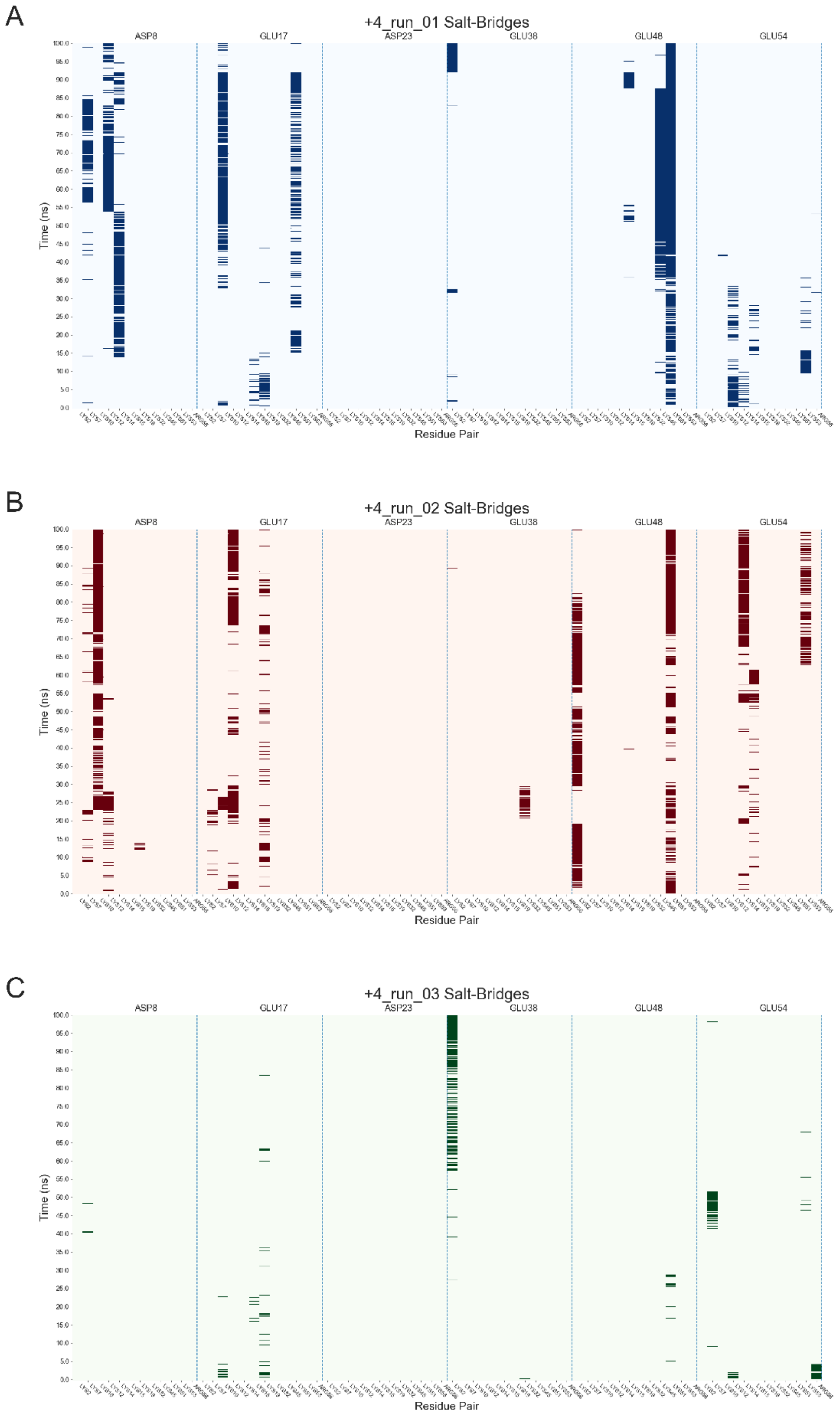

Figure S13 Salt-bridge analysis for each stable temperature, 100ns run of +4. With theoretical acidic/basic residue pairs on the x-axis, and simulation time in ns on the x-axis, with a dark = true, light = false binary for whether or not a salt-bridge is present. The acidic bases represent the 6 vertical columns, and the sub-columns represent its salt-bridge with a basic residue. The acidic

residues are, in order: ASP8, GLU17, ASP23, GLU38, GLU48, GLU54. The basic residues are, in order: LYS2, LYS7, LYS10, LYS12, LYS14, LYS15, LYS19, LYS32, LYS45, LYS51, LYS 53, ARG56. A) +4 run 1 B) +4 run 2 C) +4 run 3

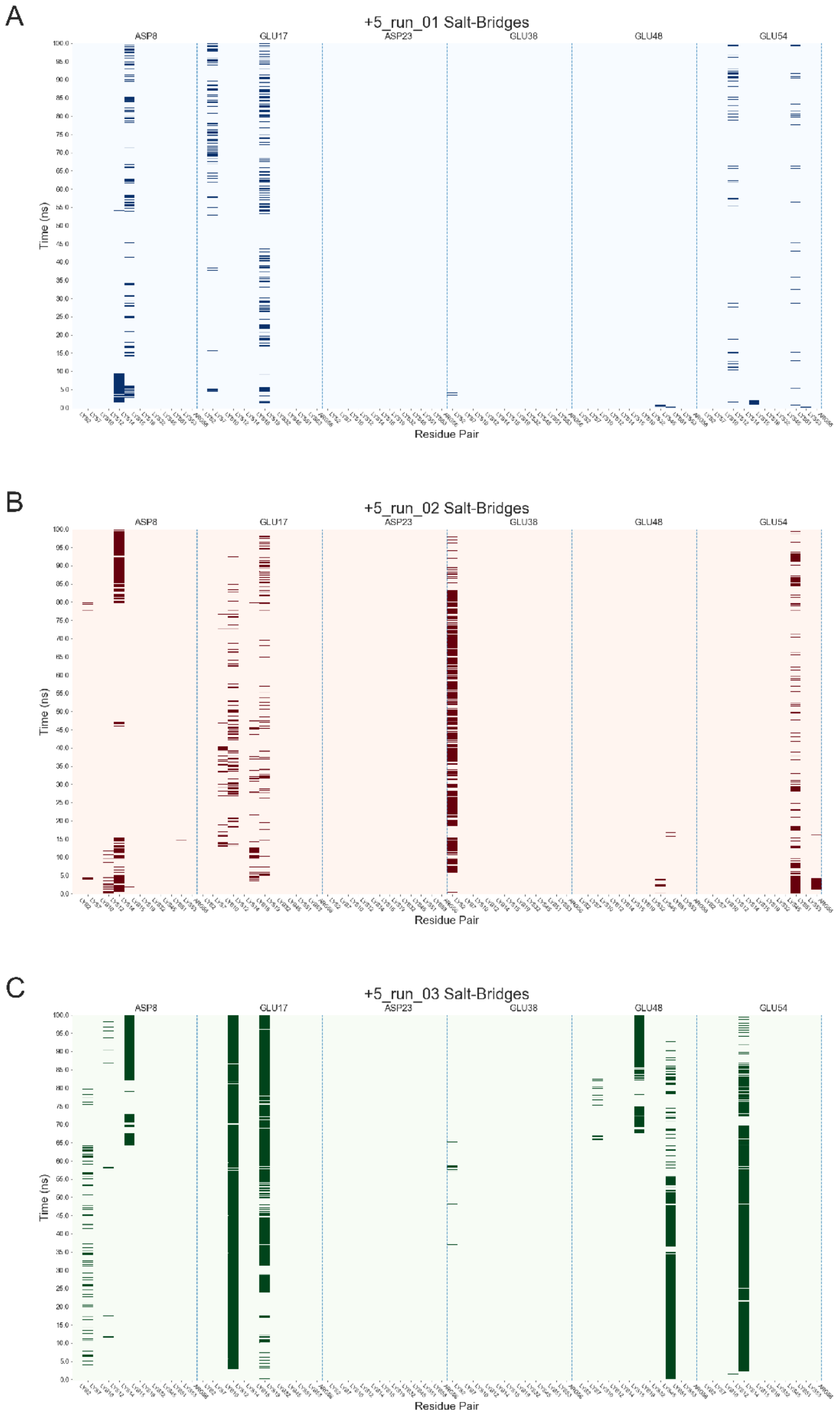

Figure S14 Salt-bridge analysis for each stable temperature, 100ns run of +5. With theoretical acidic/basic residue pairs on the x-axis, and simulation time in ns on the x-axis, with a dark = true, light = false binary for whether or not a salt-bridge is present. The acidic bases represent the 6 vertical columns, and the sub-columns represent its salt-bridge with a basic residue. The acidic

residues are, in order: ASP8, GLU17, ASP23, GLU38, GLU48, GLU54. The basic residues are, in order: LYS2, LYS7, LYS10, LYS12, LYS14, LYS15, LYS19, LYS32, LYS45, LYS51, LYS 53, ARG56. A) +5 run 1 B) +5 run 2 C) +5 run 3

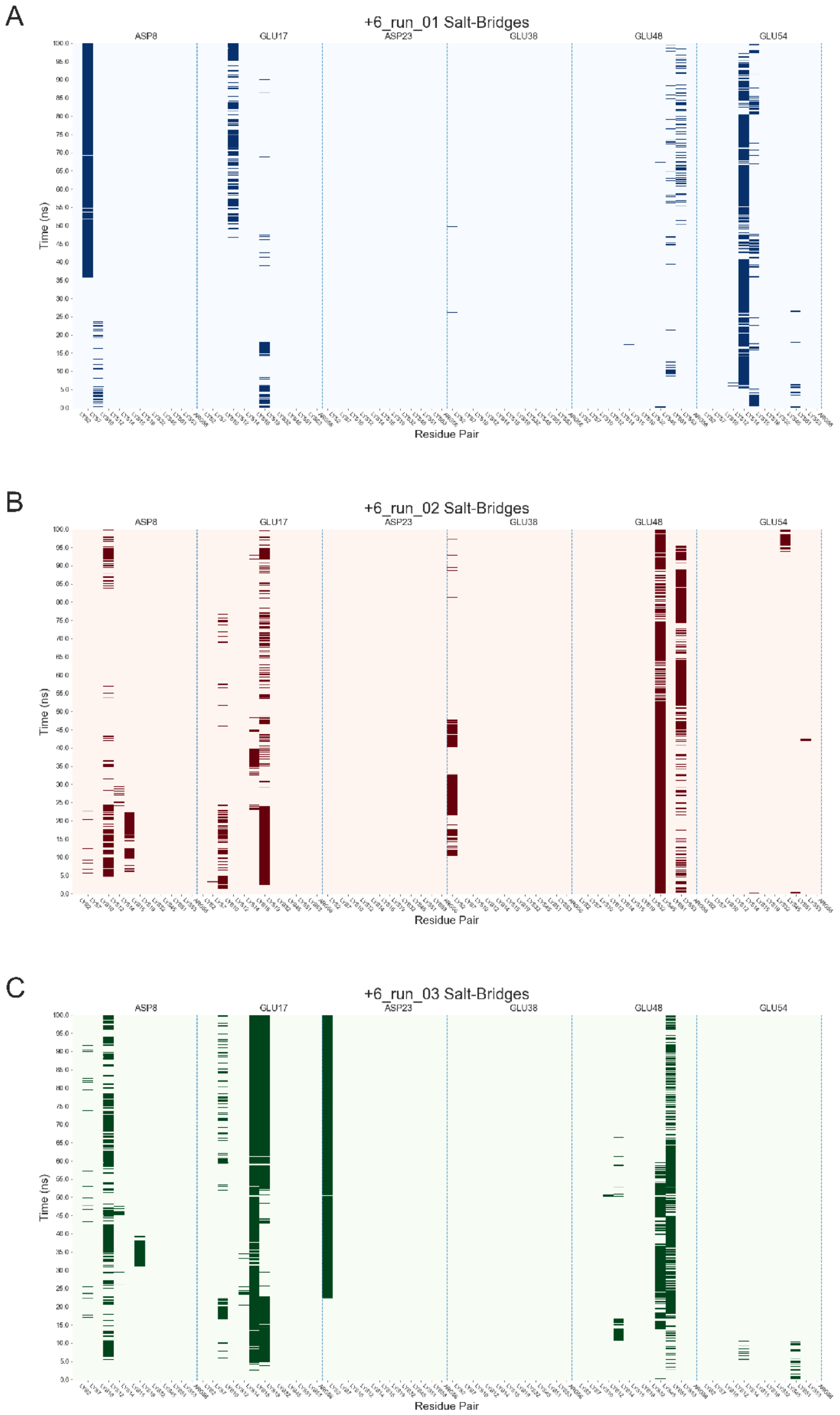

Figure S15 Salt-bridge analysis for each stable temperature, 100ns run of +6. With theoretical acidic/basic residue pairs on the x-axis, and simulation time in ns on the x-axis, with a dark = true, light = false binary for whether or not a salt-bridge is present. The acidic bases represent the 6 vertical columns, and the sub-columns represent its salt-bridge with a basic residue. The acidic

residues are, in order: ASP8, GLU17, ASP23, GLU38, GLU48, GLU54. The basic residues are, in order: LYS2, LYS7, LYS10, LYS12, LYS14, LYS15, LYS19, LYS32, LYS45, LYS51, LYS 53, ARG56/ A) +6 run 1 B) +6 run 2 C) +6 run 3

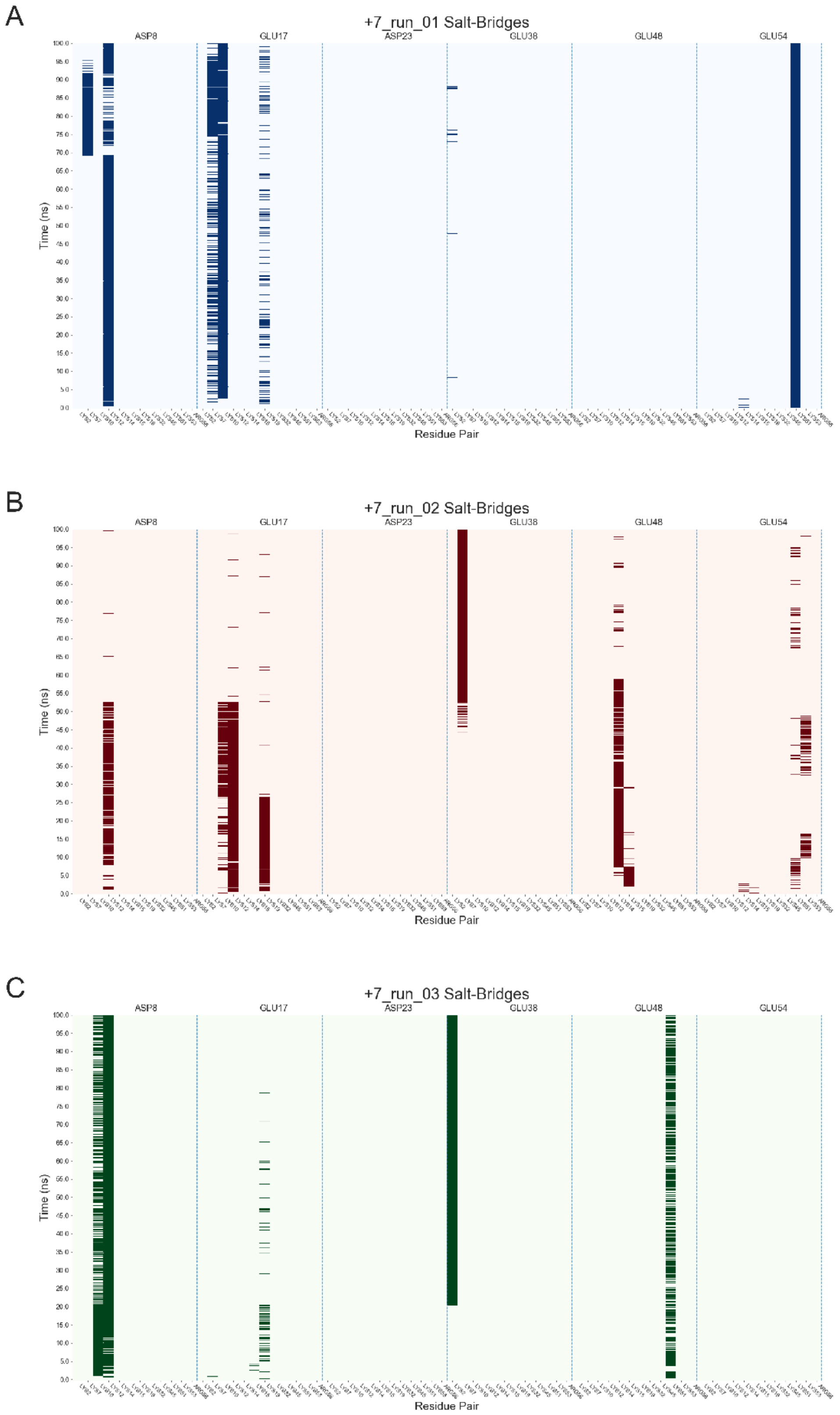

Figure S16 Salt-bridge analysis for each stable temperature, 100ns run of +7. With theoretical acidic/basic residue pairs on the x-axis, and simulation time in ns on the x-axis, with a dark = true, light = false binary for whether or not a salt-bridge is present. The acidic bases represent the 6 vertical columns, and the sub-columns represent its salt-bridge with a basic residue. The acidic

residues are, in order: ASP8, GLU17, ASP23, GLU38, GLU48, GLU54. The basic residues are, in order: LYS2, LYS7, LYS10, LYS12, LYS14, LYS15, LYS19, LYS32, LYS45, LYS51, LYS 53, ARG56. A) +7 run 1 B) +7 run 2 C) +7 run 3

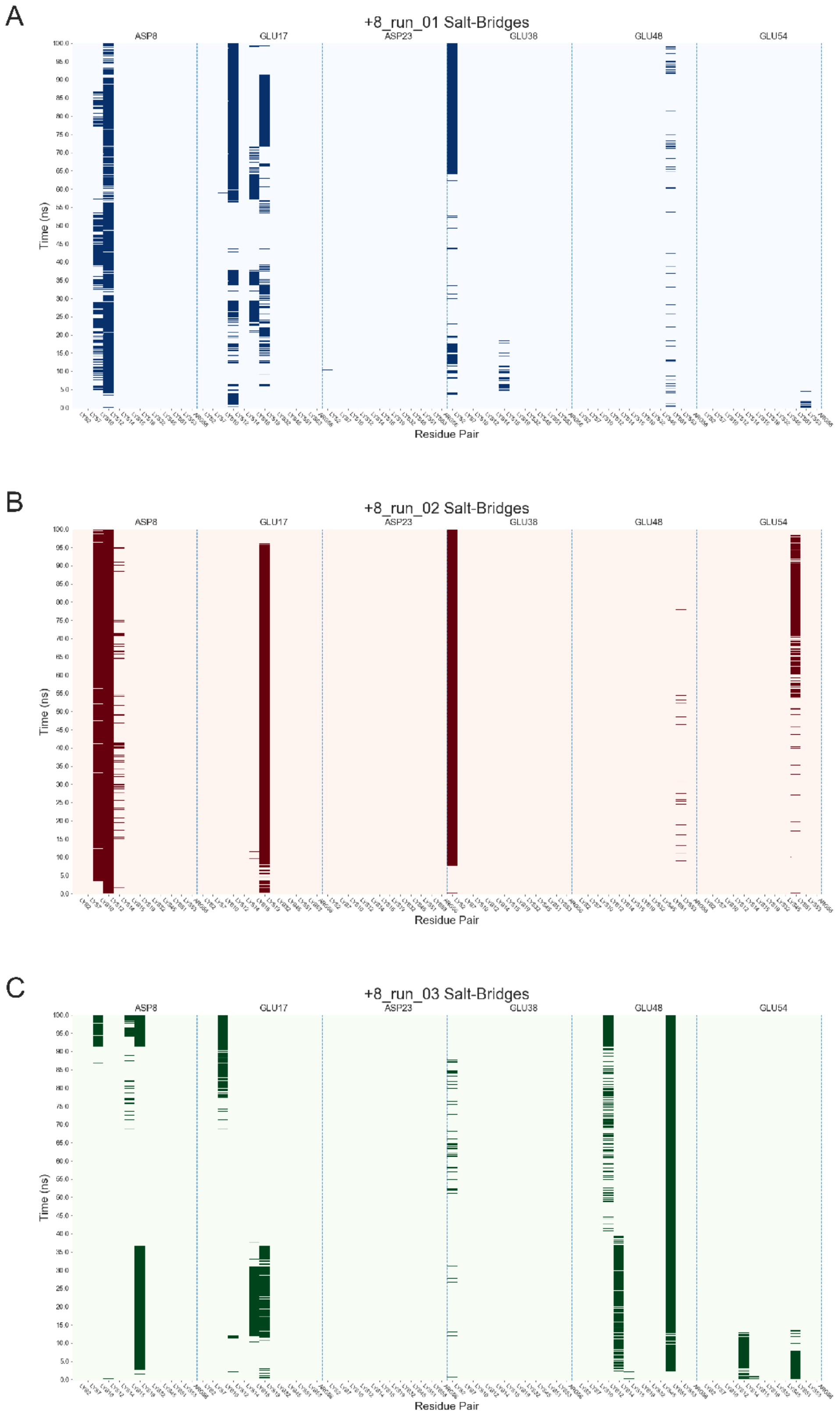

Figure S17 Salt-bridge analysis for each stable temperature, 100ns run of +8. With theoretical acidic/basic residue pairs on the x-axis, and simulation time in ns on the x-axis, with a dark = true, light = false binary for whether or not a salt-bridge is present. The acidic bases represent the 6 vertical columns, and the sub-columns represent its salt-bridge with a basic residue. The acidic

residues are, in order: ASP8, GLU17, ASP23, GLU38, GLU48, GLU54. The basic residues are, in order: LYS2, LYS7, LYS10, LYS12, LYS14, LYS15, LYS19, LYS32, LYS45, LYS51, LYS 53, ARG56. A) +8 run 1 B) +8 run 2 C) +8 run 3

Run	Impact	Collidoscope	Run	Impact	Collidoscope
1	961.457	945.849	51	908.7929	920.217
2	935.1087	921.428	52	940.2409	970.143
3	931.1575	884.251	53	967.9523	930.365
4	948.9289	941.83	54	929.2829	917.928
5	944.608	963.688	55	965.2879	963.872
6	932.0631	925.132	56	868.2057	858.471
7	905.5992	896.05	57	899.2798	874.103
8	942.671	910.936	58	907.4247	882.522
9	894.4645	910.19	59	956.5529	934.126
10	957.3677	939.652	60	889.5645	898.107
11	958.2441	898.89	61	967.9368	932.985
12	894.251	924.474	62	939.6644	966.027
13	946.4384	936.304	63	966.4752	929.681
14	872.1715	889.484	64	954.9872	915.299
15	929.3936	924.09	65	983.838	969.097
16	878.8916	860.135	66	967.5889	932.366
17	923.2822	877.922	67	995.0701	949.279
18	907.8234	913.065	68	949.8669	923.796
19	937.3027	931.502	69	980.6288	970.521
20	905.9074	889.753	70	965.2209	931.461
21	940.9813	920.745	71	950.8809	897.361
22	908.1775	925.322	72	912.9163	904.025
23	932.9945	941.266	73	1096.695	1072.34
24	965.0043	908.257	74	952.4083	949.854
25	943.2866	948.895	75	913.1128	900.747
26	942.2969	911.456	76	954.4482	929.915
27	922.3042	914.726	77	1007.683	961.715
28	942.4156	924.531	78	903.7052	896.249
29	941.831	922.826	79	978.7227	926.168
30	928.6318	923.783	80	969.5938	928.389
31	900.9894	872.453	81	886.9454	922.149
32	920.3694	906.793	82	867.8688	857.449
33	870.7073	874.254	83	920.2562	916.981
34	901.5789	900.472	84	951.4125	933.025
35	904.1796	940.723	85	954.8342	930.75
36	900.9996	930.099	86	933.2128	925.287
37	913.3879	889.822	87	856.6397	874.794
38	900.9169	933.246	88	902.3333	918.514
39	970.2994	944.639	89	936.0134	909.502
40	885.5055	903.973	90	912.6838	883.824
41	911.4626	913.431	91	941.3571	919.784
42	883.0378	892.057	92	936.6518	933.835
43	922.4573	902.513	93	938.6995	929.783
44	933.0264	921.787	94	924.8862	905.556
45	916.5276	896.588	95	922.6901	882.42
46	922.6808	910.929	96	916.553	887.871
47	900.401	899.037	97	899.4547	883.233
48	917.0013	892.211	98	925.2546	904.834
49	947.6067	932.293	99	948.3608	880.614
50	883.9801	848.727	100	1008.725	944.332

Table S8 Table of IMPACT and Collidoscope values of the final states of the +4 X100 100ns

Run	Impact	Collidoscope	Run	Impact	Collidoscope
1	1027.122	1014.2	51	967.41	955.447
2	930.1304	908.4	52	918.0463	893.642
3	1038.317	1001.8	53	950.4324	924.899
4	943.8527	919.005	54	909.1313	894.047
5	971.6848	948.986	55	985.4132	957.255
6	1045.768	1031.29	56	992.2277	966.75
7	927.824	918.324	57	907.5698	884.243
8	921.92	930.018	58	934.782	929.431
9	906.1149	913.61	59	909.2314	912.371
10	976.1089	932.493	60	944.8962	926.894
11	958.1115	925.973	61	920.1101	853.198
12	1031.823	991.462	62	978.1048	957.462
13	1026.729	991.447	63	1000.845	985.224
14	965.4467	965.85	64	1095.253	1063.9
15	919.2516	891.722	65	921.1064	907.533
16	1038.316	981.841	66	1020.144	997.856
17	971.1846	974.893	67	961.0808	947.144
18	1202.157	1144.45	68	962.7245	928.018
19	942.1443	909.733	69	993.1913	982.151
20	1072.463	1020.62	70	956.0546	934.685
21	972.0891	957.031	71	938.5427	930.131
22	1016.397	973.629	72	936.3256	917.623
23	914.6347	938.41	73	972.1666	944.451
24	973.5637	936.022	74	910.4523	942.477
25	930.4728	899.924	75	1042.945	995.919
26	884.3393	874.951	76	983.1199	953.312
27	935.435	930.667	77	970.4948	958.398
28	937.1935	943.454	78	935.3095	946.092
29	943.482	928.822	79	986.6953	957.232
30	985.4766	991.443	80	927.3837	928.836
31	917.5842	895.109	81	1038.762	999.173
32	1002.033	977.13	82	964.51	909.048
33	988.4756	958.531	83	1033.337	977.483
34	950.5987	930.075	84	951.9276	904.88
35	908.4254	925.128	85	949.5624	922.023
36	1012.85	980.458	86	981.2015	969.384
37	946.7439	906.934	87	960.4288	929.522
38	910.7776	897.222	88	937.3265	921.138
39	1004.059	956.072	89	994.481	989.424
40	959.7917	957.041	90	938.2387	914.155
41	900.6946	915.046	91	913.6196	888.562
42	932.014	932.8	92	939.7388	913.241
43	961.3199	961.627	93	984.6215	938.359
44	902.2697	917.789	94	891.2126	910.486
45	973.7716	955.614	95	944.3498	925.612
46	889.2626	905.737	96	1003.489	993.659
47	888.0189	895.206	97	976.097	926.51
48	1043.626	1037.46	98	941.6848	937.928
49	915.5615	896.92	99	950.3044	972.122
50	959.6516	955.825	100	1044.563	1007.13

Table S9 Table of IMPACT and Collidoscope values of the final states of the +5 X100 100 ns

Run	Impact	Collidoscope	Run	Impact	Collidoscope
1	1027.164	1052.28	51	964.9078	961.49
2	1006.643	983.155	52	992.4448	968.808
3	996.9927	952.678	53	914.0533	932.087
4	977.3378	960.036	54	1034.086	994.517
5	1000.104	993.296	55	1058.418	1039.19
6	1010.547	963.002	56	1050.84	1007.72
7	1078.805	1021.46	57	965.9869	948.34
8	1010.303	997.566	58	1064.732	1079.6
9	1040.259	1043.95	59	1025.012	983.512
10	1023.466	990.023	60	994.4432	952.194
11	1042.101	1018.36	61	1079.195	1036.54
12	1015.358	1004.96	62	963.9572	939.801
13	1094.281	1020.2	63	1025.587	985.767
14	1063.135	1063.52	64	1035.459	1003.31
15	1114.347	1074.3	65	964.0439	953.166
16	969.4725	984.734	66	931.91	971.571
17	1027.779	1054.83	67	1024.295	989.974
18	1008.376	1003.55	68	1067.277	1039.3
19	1040.529	1027.67	69	1000.527	959.833
20	979.4949	973.501	70	1010.979	1003.58
21	990.0956	965.992	71	998.9255	991.737
22	1017.791	993.849	72	991.145	973.619
23	1077.131	1029.43	73	1020.646	992.025
24	964.1937	919.689	74	1180.493	1113.65
25	1029.727	990.952	75	961.7761	932.202
26	1147.095	1098.19	76	1027.114	1003.68
27	1043.853	1053.28	77	999.5484	999.113
28	992.0992	969.422	78	951.2872	947.448
29	976.0408	961.515	79	1019.787	1007.36
30	1007.635	972.381	80	1006.999	959.825
31	979.334	950.432	81	1119.764	1031.53
32	1109.068	1102.58	82	984.5826	978.276
33	1071.528	1025.58	83	943.9882	939.154
34	1013.742	992.506	84	998.0789	962.905
35	1013.762	986.416	85	1025.522	997.366
36	998.5925	985.719	86	973.9164	966.076
37	1095.625	1038.97	87	1082.75	1063.36
38	999.6476	975.564	88	965.2268	951.637
39	1009.28	1038.48	89	1014.934	999.767
40	1053.664	1026.3	90	997.7878	977.214
41	1018.393	1001.85	91	960.4033	931.451
42	1044.012	1017.64	92	1005.762	983.996
43	1028.042	1004.78	93	1012.448	997.066
44	1008.887	991.487	94	985.8418	977.628
45	1047.659	1043.83	95	1020.442	1006.27
46	1031.137	1028.54	96	982.7318	960.661
47	1028.753	1010.54	97	975.513	942.292
48	1034.416	1020.02	98	1114.979	1125.56
49	1100.861	1084.14	99	978.2052	969.928
50	1021.276	967.078	100	963.3795	924.81

Table S10 Table of IMPACT and Collidoscope values of the final states of the +6 X100 100 ns

Run	Impact	Collidoscope	Run	Impact	Collidoscope
1	1042.003	1048.28	51	1047.906	1015.81
2	1194.381	1142.48	52	1105.635	1055.95
3	1380.361	1312.95	53	1151.645	1108.48
4	1381.441	1348.47	54	1259.603	1152.6
5	1028.765	995.532	55	1049.121	1004.31
6	1038.582	1001.09	56	1036.822	1013.47
7	1066.581	1069.64	57	1212.774	1222.35
8	1008.547	1010.4	58	977.8166	985.304
9	1046.714	1041.36	59	1060.242	1029.94
10	988.1608	978.777	60	1166.481	1132.08
11	1177.381	1104.63	61	973.8894	942.847
12	1059.503	1033.31	62	986.7734	938.191
13	1028.594	1002.68	63	1222.284	1126.98
14	1300.544	1281.79	64	1095.596	1037.61
15	1071.653	1054.34	65	1154.726	1057.74
16	1341.769	1238.53	66	1180.908	1147.72
17	986.2558	971.683	67	1040.62	1019.37
18	1082.849	1105.15	68	1241.076	1148.22
19	1216.825	1113	69	1021.104	994.528
20	979.9822	986.076	70	973.7582	948.091
21	1225.804	1126.15	71	1248.433	1168.23
22	1004.367	979.53	72	1193.296	1110.69
23	1019.727	1011.82	73	988.9558	960.056
24	1058.814	1045.01	74	1032.57	994.116
25	1227.395	1145.25	75	1202.734	1167.31
26	1037.021	1047.35	76	988.0106	1017.21
27	1104.49	1078.45	77	1367.253	1238.06
28	987.845	1007.1	78	1104.785	1105.79
29	1169.986	1079.94	79	1001.582	1021.48
30	951.6939	987.959	80	1146.716	1144.66
31	1026.663	1020.35	81	1057.937	997.139
32	1216.048	1176.51	82	1072.721	1066.28
33	976.8174	971.835	83	1254.588	1180.15
34	1211.929	1137.18	84	1032.701	1019.65
35	1009.483	986.861	85	994.0247	972.258
36	1026.513	1014.77	86	986.1334	944.885
37	1180.611	1099.36	87	1035.985	1045.55
38	1226.428	1163.64	88	1020.821	1060.96
39	1027.621	1033.65	89	1024.255	1029.54
40	1014.404	1004.59	90	1029.921	999.25
41	998.8715	999.372	91	1133.759	1076.48
42	1011.342	994.295	92	1046.425	1015.46
43	1034.091	1013.46	93	1045.076	1036.47
44	1067.469	1075.64	94	1204.589	1091.04
45	1044.475	1047.87	95	1126.142	1159.18
46	1155.427	1152.26	96	1142.872	1102.63
47	983.6904	1006.94	97	1062.807	1042.49
48	1021.167	978.586	98	1116.599	1085.16
49	1012.116	1015.11	99	1024.637	1002.31
50	1091.848	1087.43	100	1142.866	1102.86

Table S11 table of IMPACT and Collidoscope values for the final states of the +7 x100 100 ns

Run	Impact	Collidoscope	Run	Impact	Collidoscope
1	1085.511	1049.95	51	1009.863	1001.64
2	1039.068	1048.2	52	1247.252	1137
3	1274.635	1154.79	53	1278.164	1174.63
4	1130.035	1125.98	54	1355.694	1162.18
5	1153.747	1101.37	55	1029.04	1070.34
6	1271.765	1103.04	56	1240.439	1187.39
7	1061.464	1088.56	57	1301.304	1104.33
8	1300.473	1168.36	58	1108.277	1056.95
9	1044.475	1035.62	59	1185.723	1188.27
10	1300.767	1189.21	60	1166.523	1156.46
11	1109.193	1115.1	61	1190.925	1125.08
12	1041.036	1071.5	62	1117.898	1050.52
13	1158.518	1113.75	63	1049.034	1055.46
14	1138.759	1088.78	64	1253.42	1098.74
15	1044.227	1040.09	65	1339.177	1218.75
16	1038.92	1022.91	66	1228.253	1243.7
17	1214.645	1178.62	67	1407.204	1274.1
18	1340.874	1193.21	68	1052.384	1044.02
19	1064.362	1045.03	69	1170.507	1109.94
20	1264.241	1152.69	70	1300.029	1283.4
21	1255.185	1112.58	71	1005.149	1040.83
22	1046.343	1063.48	72	1131.744	1116.25
23	1085.72	1057.47	73	1411.643	1360.66
24	1104.133	1100.19	74	1064.465	1091.13
25	1122.196	1076.87	75	1049.353	1052.25
26	1057.432	1057.87	76	1167.745	1122.4
27	1166.094	1106.7	77	1084.488	1063.76
28	1305.198	1126.7	78	1234.411	1159.61
29	1222.705	1184.86	79	1278.158	1117.94
30	1172.824	1163.96	80	1087.835	1123.55
31	1060.73	1090.62	81	1305.171	1169.88
32	1088.107	1047.6	82	1071.061	1015.19
33	1121.777	1100.59	83	1305.517	1169.07
34	1217.707	1188.75	84	1096.42	1070.4
35	1040.609	1039.36	85	1064.882	1015.74
36	1335.553	1209.63	86	1324.358	1251.18
37	1088.555	1039.64	87	1050.02	1049.52
38	1080.039	1076.26	88	1174.723	1143.42
39	1088.146	1078.63	89	1316.208	1175.39
40	1090.702	1084.44	90	1326.745	1206.96
41	1118.745	1098.52	91	1082.206	1045.08
42	1152.985	1138.35	92	1299.301	1182.36
43	1271.948	1245.21	93	1095.512	1093.11
44	1053.561	1073.09	94	1099.773	1054.7
45	1094.404	1089.63	95	1147.446	1124.61
46	1117.17	1077.89	96	1282.805	1242.54
47	1139.745	1087.81	97	1015.423	1022.67
48	1222.658	1158.16	98	1070.602	1061.34
49	1038.037	1035.91	99	1088.168	1071.78
50	1040.971	1040.76	100	1081.519	1038.62

Table S12 Table of IMPACT and Collidoscope values of the final states of the +8 X100 100 ns

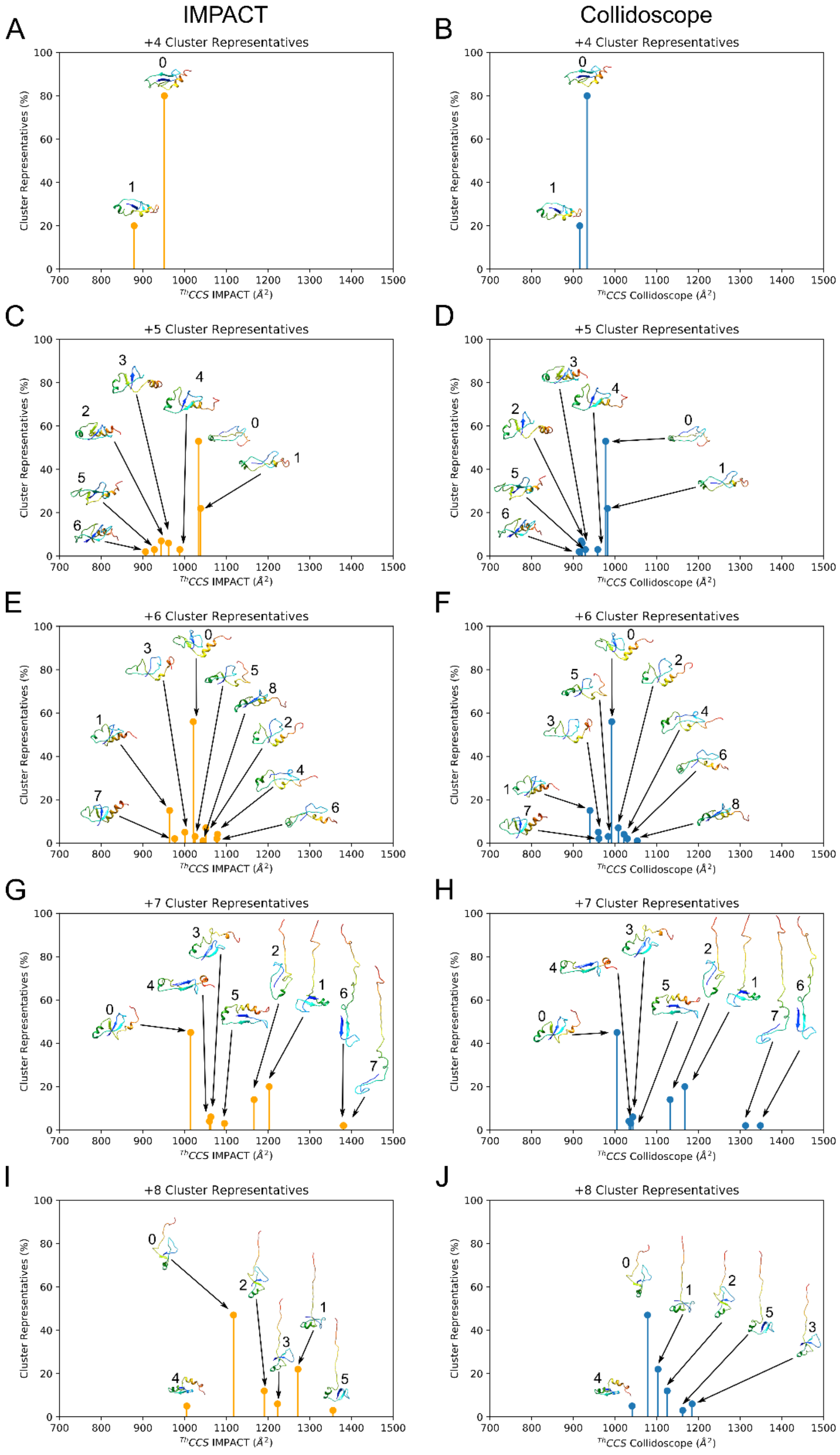

Figure S18: **:** Ensemble cluster analysis of the 100 outcomes of the coulombic unfolding simulations, with the THCCS on the x-axis and the percentage of structures represented on the y-axis. Each cluster has the representative structure shown above, with its corresponding rank. These structures represent ≥95% of outcomes. For full data please see table S13 Structures with **A, C, E, G I)** IMPACT THCCS **B, D F, H, J)** Collidoscope THCCS **A-B)** +4, **C-D)** +5, **E-F)** +6, **G-H)** +7 and **I-J)** +8 simulations.

+z	rank	Percent (%)	Run model	IMPACT (Å^2)	Collidoscope (Å^2)
+4	0	80	084	951.4125	933.025
	1	20	016	878.8916	860.135
+5	0	53	083	1033.337	977.483
	1	22	016	1038.316	981.841
	2	7	004	943.8527	919.005
	3	6	043	961.3199	961.627
	4	3	033	988.4756	958.531
	5	3	080	927.3837	928.836
	6	2	009	906.1149	913.61
+6	0	56	073	1020.646	992.025
	1	15	062	963.9572	939.801
	2	7	056	1050.84	1007.72
	3	5	069	1000.527	959.833
	4	4	007	1078.805	1021.46
	5	3	059	1025.012	983.512
	6	2	023	1077.131	1029.43
	7	2	029	976.0408	961.515
	8	1	027	1043.853	1053.28
+7	0	45	40	1014.404	1004.59
	1	20	75	1202.734	1167.31
	2	14	60	1166.481	1132.08
	3	6	97	1062.807	1042.49
	4	4	12	1059.503	1033.31
	5	3	64	1095.596	1037.61
	6	2	4	1381.441	1348.47
	7	2	3	1380.361	1312.95
+8	0	47	046	1117.17	1077.89
	1	22	006	1271.765	1103.04
	2	12	061	1190.925	1125.08
	3	6	029	1222.705	1184.86
	4	5	071	1005.149	1040.83
	5	3	054	1355.694	1162.18

Table S13: Ensemble cluster analysis outcomes of the 100ns coulombic simulations, showing the charge state, the rank of each cluster, the % of structures that cluster represents, the run number that is representative of the cluster and the THCCS of the structure by IMPACT and Collidoscope.
